# Eliciting patient views on the allocation of limited healthcare resources: a deliberation on hepatitis C treatment in the Veterans Health Administration

**DOI:** 10.1186/s12913-020-05211-8

**Published:** 2020-05-01

**Authors:** Akbar K. Waljee, Kerry A. Ryan, Chris D. Krenz, George N. Ioannou, Lauren A. Beste, Monica A. Tincopa, Sameer D. Saini, Grace L. Su, Maria E. Arasim, Patti T. Roman, Brahmajee K. Nallamothu, Raymond De Vries

**Affiliations:** 1 VA Ann Arbor Health Services Research and Development Center of Clinical Management Research, 2215 Fuller Road, Mail Stop 152, Ann Arbor, MI 48105 USA; 2 Michigan Medicine, Department of Internal Medicine, Division of Gastroenterology and Hepatology, 3912 Taubman Center, 1500 East Medical Center Drive, SPC 5362, Ann Arbor, MI 48109-5362 USA; 3 Michigan Integrated Center for Health Analytics and Medical Prediction (MiCHAMP), 2800 Plymouth Road, North Campus Research Complex (NCRC), Building 16, Ann Arbor, MI 48109-2800 USA; 4 Center for Bioethics and Social Sciences in Medicine, University of Michigan Medical School, 2800 Plymouth Road, North Campus Research Complex, Bldg. 14, G016, Ann Arbor, MI 48109-2800 USA; 5 Veterans Affairs Puget Sound Healthcare System, 1660 South Columbian Way, Seattle, WA 98108 USA; 6 Division of Gastroenterology, Department of Medicine, University of Washington, 1959 NE Pacific St., Box 356424, Seattle, WA 98195-6424 USA; 7 Division of General Internal Medicine, University of Washington, Harborview Medical Center, 325 Ninth Ave, Box 359780, Seattle, WA 98104 USA; 8 Michigan Medicine, Department of Internal Medicine, Division of Cardiovascular Medicine, 1500 East Medical Center Drive, SPC 5856, Ann Arbor, MI 48109-5362 USA

**Keywords:** Veterans, VA, Public deliberation, Democratic Deliberation, Hepatitis C, Qualitative research, Health policy

## Abstract

**Background:**

In response to the development of highly effective but expensive new medications, policymakers, payors, and health systems are considering novel and pragmatic ways to provide these medications to patients. One approach is to target these treatments to those most likely to benefit. However, to maximize the fairness of these policies, and the acceptance of their implementation, the values and beliefs of patients should be considered. The provision of treatments for chronic hepatitis C (CHC) in the resource-constrained context of the Veterans Health Administration (VHA) offered a real-world example of this situation, providing the opportunity to test the value of using Democratic Deliberation (DD) methods to solicit the informed opinions of laypeople on this complex issue.

**Methods:**

We recruited Veterans (*n* = 30) from the VHA to attend a DD session. Following educational presentations from content experts, participants engaged in facilitated small group discussions to: 1) identify strategies to overcome CHC treatment barriers and 2) evaluate, vote on, and modify/improve two CHC treatment policies – “first come, first served” (FCFS) and “sickest first” (SF). We used transcripts and facilitators’ notes to identify key themes from the small group discussions. Additionally, participants completed pre- and post-DD surveys.

**Results:**

Most participants endorsed the SF policy over the FCFS policy, emphasizing the ethical and medical appropriateness of treating the sickest first. Concerns about SF centered on the difficulty of implementation (e.g., how is “sickest” determined?) and unfairness to other Veterans. Proposed modifications focused on: 1) the need to consider additional health factors, 2) taking behavior and lifestyle into account, 3) offering education and support, 4) improving access, and 5) facilitating better decision-making.

**Conclusions:**

DD offered a robust and useful method for addressing the allocation of the scarce resource of CHC treatment. Participants were able to develop a modified version of the SF policy and offered diverse recommendations to promote fairness and improve quality of care for Veterans. DD is an effective approach for incorporating patient preferences and gaining valuable insights for critical healthcare policy decisions in resource-limited environments.

## Contributions to the literature

- There is growing interest in incorporating the patient voice in the development of healthcare policy.
- This study confirms the value of Democratic Deliberation (DD) for eliciting the informed preferences of Veterans regarding the allocation of limited Chronic Hepatitis C (CHC) treatment resources.
- In the case of CHC treatment, Veterans generally preferred a “sickest first” approach over a “first come, first served” approach.
- Our research has implications for treatment allocation policies in resource-limited settings beyond the VHA. Moreover, the approach of using DD to incorporate the opinions of patients has implications for how we develop healthcare policy for ethically controversial issues.

## Background

The Veterans Health Administration (VHA) is an integrated health system responsible for providing healthcare to over 9 million Veterans [1]. Given its large number of clients and its finite funding, the VHA has implemented multiple programs and policies to address the problem of resource limitations [2]. The success of these policies is evidenced by the fact that the VHA outperforms the private sector on many health-related outcomes [3–5]. Among the many challenges facing the VHA was the question of how to provide access to the effective, but expensive, direct acting antiviral treatment for chronic hepatitis C virus (CHC) infection [6]. Like other healthcare providers, including health maintenance organizations and state-funded Medicaid programs, the VHA is grappling with the problem of finding ways to provide this and other new and costly medications to its patients.

One approach is to target these costly treatments to those most likely to benefit, using quantitative prediction models to estimate disease progression or likelihood of response to therapy. The use of this strategy for the allocation of CHC treatment benefit seems promising, but to maximize the fair use of this approach and likely acceptance of its implementation, the values and beliefs of patients should be considered.

There is growing interest in incorporating the patient voice in healthcare policies regarding ethically controversial issues that impact health [7–12]. Because complex healthcare decisions tend to require a sophisticated level of knowledge and are often emotionally charged, traditional opinion surveys are of little value. Specifically, survey respondents frequently lack a nuanced understanding of the complexities and the underlying ethical challenges, and thus surveys can provide only superficial insights into a population’s policy preferences [12]. On the other hand, qualitative approaches – interviews and focus groups – provide richer data than surveys but are of limited value if the participants remain uninformed. To address these problems, we used Democratic Deliberation (DD) methods as a proof of concept for informing policy decisions related to the allocation of scarce resources. Democratic Deliberation methods have proven useful for soliciting patient opinions in other contexts [11, 12]; in this study we examine the value of DD for learning and incorporating the opinions of Veterans about the use of health system resources.

## Methods

Democratic Deliberation methods combine education by experts with guided discussion among peers to deliver informed opinions and suggestions to policymakers and other concerned stakeholders. It is a cooperative process that allows participants to respond to, and build on, the interests and perspectives of their fellow deliberators [13–16]. The deliberative process generates policy recommendations, as well as the rationales underlying those recommendations. Democratic Deliberation provides a practical and reliable approach for soliciting the informed and considered opinions of lay participants, empowering them to shape policy on complex issues [17].

### Recruitment

Recruitment for the DD session targeted Veterans with and without CHC from the VA Ann Arbor Healthcare System (VAAAHS) including affiliated community-based outpatient clinics. Eligible participants had at least one visit to a VHA facility within the previous 12 months, lived within 60 miles of the VAAAHS, and agreed to have their voices recorded during the DD session. We stopped recruitment after consenting 37 Veterans, 30 of whom ultimately attended the deliberation. This allowed us to have 6 small groups with an average of 5 participants each, which was an appropriate number given the venue and, in our experience, is enough participants to generate substantive discussion while still being manageable for the facilitators and presenters. To provide a broad perspective, the 37 Veterans who consented to participate were stratified based on race, gender, and CHC diagnosis. Each of the 30 (81%) participants who attended the DD session received a $200 incentive in appreciation for their time and to offset transportation costs.

### DD procedures and materials

#### General overview

Based on a model used in previous studies, participants took part in a 6-h DD session comprised of educational presentations, facilitated small group discussions, a larger plenary discussion, and pre- and post-DD surveys [15, 18]. Each element is discussed in greater detail below. Table 1 contains a more detailed agenda.

**Table 1:** Deliberation Agenda

Time	Activity & Topic
9:00–9:30 (30 min)	**Registration** ● Continental breakfast, informed consent, and Baseline Survey
9:30–9:45 (15 min)	**Welcome** ● Presentation on Deliberative Democracy: Why are we here? What are we going to do? What is Deliberative Democracy?
9:45–9:55 (10 min)	**Introductions (Small Groups)**
9:55–10:25 (30 min)	**Large Group Session ONE** ● Presentation on the Hepatitis C Virus and its Treatment, Q&A
10:25–11:10 (45 min)	**Small Group Session ONE** ● Discussion of the presentation and brainstorming other barriers to treating Veterans and ways to overcome those barriers.
11:10–11:55 (45 min)	**Lunch**
11:55–12:55 (60 min)	**Large Group Session TWO** ● Presentations on: ○ Caring for Veterans with Hep-C in the VA, Q&A ○ “First Come, First Served” and “Sickest First” Policies, Q&A
12:55–1:50 (55 min)	**Small Group Session TWO** ● Part 1: Discussion on the Pros and Cons of each policy, followed by an Anonymous Vote for their preferred policy. ● Part 2: Brainstorming ideas for modifying/improving the policies.
1:50–2:00 (10 min)	**Break**
2:00–2:30 (30 min)	**Plenary Discussion** ● Each facilitator reports their group’s preferred policy and reasoning. ● Room-wide discussion of the policies.
2:30–3:00 (30 min)	**Post-Deliberation Survey** ● Final Survey and compensation.

The study team consulted with several DD and CHC experts in developing the DD session agenda, surveys, and discussion materials to provide a comprehensive overview of CHC and the challenges that stakeholders face when creating policies for such chronic diseases as CHC. We also conducted preliminary interviews with 11 Veterans (with and without CHC) to guide development of these materials (see Additional file 3 for the interview guide). The DD was held in July of 2018.

#### Surveys

Each Veteran completed a survey at the beginning and end of the DD session to measure their experience, knowledge, beliefs, and preferences regarding CHC and its treatment. These data are summarized below and in Additional file 1. Given the small sample size, we were unable to evaluate the data for statistically significant associations. This manuscript focuses on a qualitative analysis of the small group discussions to identify important themes. The survey instruments for the baseline and follow-up surveys are in Additional files 4 and 5.

#### Expert presentations

The presentations were given by four physician experts who provided education on the liver, CHC (including routes of transmission, such as drug use), symptoms, treatment (including the recent and rapid improvements in their efficacy), and a history of CHC treatment policies. Each presentation ended with a brief Q and A period and provided context to the small group session that followed. The experts were also available during the small group discussions to answer any additional questions. The experts and facilitators included both women and men with a variety of racial/ethnic backgrounds.

#### Small group discussions

Each participant was assigned to one of six small groups led by a trained facilitator. Five of the six groups had a mix of Veterans with and without CHC, and the remaining group had all Veterans with CHC. Small group facilitators were selected based on experience in qualitative interviewing and/or facilitation. Prior to the DD session, facilitators attended training sessions that provided an overview of the DD session agenda, education on the research topic, and a review of small group activities they would be leading.

The first small group deliberation asked Veterans to brainstorm possible barriers to obtaining CHC treatment and ideas about ways to overcome these barriers. The second small group session asked Veterans to discuss and vote on two specific policies: 1) treat Veterans with CHC on a first come, first served (FCFS) basis policy or 2) treat Veterans with CHC who are sickest first (SF) policy. After voting, the small groups then discussed ways to modify or improve upon their preferred policy. The facilitator and participant discussion packets can be found in Additional files 6 and 7.

#### Plenary discussion

The day concluded with a plenary session where each group presented their preferred policy to the larger group, who then reacted and asked questions. This session was moderated by a DD expert ensuring that all participants had the opportunity to share their viewpoint with the entire group.

### Small group discussion analysis

Facilitators took notes during the discussion and wrote the Veterans’ ideas on a large paper easel to guide the conversation. Presentations and group discussions were recorded, transcribed, and de-identified with participants’ consent. After the DD session, facilitator notes were analyzed for main themes across small groups and these were used as preliminary thematic domains. These domains were then further confirmed and refined through a thematic analysis of the transcripts, led by KR. Relevant quotes were identified that captured the reasoning used by Veterans to support preferences for treatment models. Themes and supporting quotes were reviewed multiple times to ensure accuracy.

The Institutional Review Board at the VAAAHS gave approval for this study, and participants provided written informed consent. We followed the Standards for Reporting Quality Research guidelines [19].

## Results

The average age of attendees was 61.0 years (SD 8.7), 11 (37%) were female, 15 (50%) identified as non-Hispanic white, and 18 (60%) had current or treated CHC. A majority (83%) said they are “Satisfied” or “Very Satisfied” with the care they receive at the VHA. Additional demographics are presented in Table 2.

**Table 2:** Deliberation Participant Characteristics (*n* = 30)

	n (%)^**a**^
**Gender**	
Female	11 (37)
Male	19 (63)
**Age, Mean (SD)**	61 (9)
**Race**	
White	15 (50)
Black	10 (33)
Other	5 (17)
**Education**	
High School Diploma/GED or Less	10 (33)
Some College or 4-Year College Degree	16 (53)
More than 4-Year College Degree	4 (13)
**Marital Status**	
Married or Living with Partner	14 (48)
Divorced, Separated, or Widowed	11 (38)
Never Married	4 (14)
**Annual Household Income**^**b**^	
$5000 - $19,999	10 (37)
$20,000 - $39,999	8 (30)
$40,000 or Higher	9 (33)
**Employment**	
Working Full or Part-time	8 (29)
Unemployed, Retired, or Disabled	18 (64)
Other	2 (7)
**Branch of Military Service**	
Army	19 (63)
Navy	10 (33)
**Era of Military Service**	
Vietnam Era (1964 to 1975)	15 (52)
Late/Post Cold War Era (1976 to 2001)	11 (38)
War on Terrorism (2001 to present)	3 (10)
**Length of Service (Years), Mean (SD)**	6 (5)
**Where Veteran receives most of their care**	
VA facility	25 (89)
Non-VA facility	3 (11)

^a^Valid percentages of non-missing data are shown

^b^Annual Household Income is collapsed from 20 categories

Although both the survey and presentations specified that resources are limited, some participants did not accept this excuse for delaying Veterans’ treatment. When given the option in the survey, almost two thirds (64% before; 62% after) of participants insisted that all Veterans be treated without delay regardless of symptoms or degree of disease severity. Only when required to choose between two policies—“first come, first served” and “sickest first”—did a majority opt for the “sickest first” policy (86% before; 93% after). In addition, a majority said they “agree” or “strongly agree” that they trust their VHA healthcare *system* (60% before; 57% after) or individual VHA healthcare *providers* (63% before and after) to decide which Veterans with CHC get treated first. Out of 7 true/false knowledge questions about CHC, Veterans answered an average of 5.4 correctly before the deliberation and 6.4 after, suggesting the educational component did have some effect. Additionally, on a scale of 1 (not at all) to 10 (very much), participants felt that their opinions were respected by the group (8.9), they were listened to by their facilitator (9.5), and that the process that led to their group’s decision was fair (9.2). Additional details are presented in Additional file 1.

### Barriers to CHC treatment

Small group discussions were guided by facilitators as needed but were primarily participant-led, with discussants posing questions to one another and reacting to each others’ comments. Although the six small groups worked independently, there was a great deal of convergence on the types of CHC treatment barriers identified. Themes that were common to several groups included: a) lack of knowledge about CHC and its treatment, b) difficulties associated with getting tested for CHC and following through with CHC treatment, c) the stigma often associated with CHC and the effect of that stigma on members of vulnerable populations, d) lack of transportation and other difficulties accessing healthcare, including limitations posed by insufficient funding and other resources within the VHA. We offer examples of comments in each of these areas below. Additional examples can be found in Additional file 2.

#### Lack of knowledge

Participants felt that a lack of knowledge and misconceptions about CHC and its treatment were obstacles to getting care. For example, participants pointed out that Veterans may not know they have hepatitis C or may not understand the seriousness of the disease.

*Participant-103: … I would think the fact that some people don’t experience any symptoms would be a barrier, so they don’t even know that they have it.*

*Participant-112: … Basically, most of it is, actually is all awareness of hepatitis C. I think there’s [...] a lot of people that really doesn’t know the severity of hepatitis C...*

#### Getting tested for CHC and adhering to treatment

Participants identified a variety of reasons that make it difficult to get some Veterans to seek testing or treatment. These reasons include fear, distrust, and unwillingness (or inability) to either seek testing or adhere to treatment.

For example, participants pointed out that Veterans may be afraid to learn they have CHC.

*Participant-110: Some people are like, “I’m not going. I don’t even want to know. I don’t want to know if I have this or not. I don’t even care how easy it is to cure it. I don’t care if I have it because what does that mean if I find out I have it?”*

*Participant-109: Yeah, it’s easier to be in denial.*

Participants added that some Veterans distrusted the healthcare system.

*Participant-105: I guess that just brings to mind just an individual having a lack of trust about the treatment maybe because they have some side effects they don’t like or for any number of reasons.*

*Participant-110: Don’t trust the VA at all because they think they got it [*i.e.*, hepatitis C] from the Army.*

Participants also pointed out that some Veterans find it difficult to follow the treatment regimen.

*Participant-123: We had people dropping out. One guy dropped out. He didn’t want to quit drinking. The other people dropped out because they didn’t have transportation. We had one person dropped out, just refused to keep taking it on time, you know, you get up every morning and you take the pill.*

#### Vulnerable populations and stigma

Vulnerable populations – patients suffering from mental health issues, addiction, or homelessness – experience additional barriers to pursuing CHC treatment.

For example, participants discussed the particular challenge of getting people living with dependency disorder to seek treatment.

*Participant-115: Somebody who shoots up drugs is going to have to first want to be able to get rid of the addiction or get clean and if he doesn’t want to get clean, then he’s not going to be interested in a trial program or whatever, getting help.*

Participants pointed out that homeless Veterans also find it difficult to seek out treatment.

*Participant-118: If you’re homeless you don’t have an address so you ain’t getting nothing …*

*Participant-115: ... if you’re homeless, you probably also don’t have access to healthcare, even getting a way to the VA.*

These vulnerable populations are also more likely to experience the burden of shame and stigma, which may prevent them from seeking treatment.

*Participant-102: I can see that or IV drug use, same thing.*

*Participant-128: Stigma.*

*Participant-101: Yeah, you want to hide, conceal, say, “So I’m not going to tell anybody, I’m going to die of hepatitis C and liver cirrhosis.”*

*Participant-101: Is shame a possible thing that we wouldn’t, you’d ignore your symptoms and wouldn’t talk to a doctor because you don’t want anybody knowing what your past … possibly?*

#### Transportation, access to treatment and cost of treatment

Access to care was another significant barrier identified by participants. Lack of access was seen not only as the result of the difficulty in finding transportation to the VA, but also a consequence of insufficient VA staff for treating CHC patients.

*Participant-126: A lot of people don’t have rides. That’s just a simple thing.*

*Participant-118: … the doctors are already so overloaded that’s why you only get 15 min sessions, so I think it would be difficult [ … ] the barriers and things of hiring people, the cost, and the VA just doesn’t have time or money to chase down somebody who’s not going to make those efforts.*

Some participants expressed frustration with government cuts and the inadequate funding for the VA that serve as barriers to access, given the sacrifices Veterans made for their country.

*Participant-123: People went out there and served our country. We ought to be able to be transported back and forth to the hospitals and you know they’re cutting money every time from the VA.*

*Participant-122: So, by putting our lives on the line, I don’t really accept someone telling me it costs too much. I think that’s an insult [ … ] I mean if a person needs to be tested for hepatitis C and it costs $10,000, you know what, this guy was going to die for us, for our nation.*

### The policy preferences of Veterans: sickest first or first come, first served?

During the small group sessions, most participants (22 participants, 73.3%) endorsed SF policy over FCFS policy. Three small groups unanimously endorsed SF policy and three were split between SF and FCFS policy.

#### Sickest first policy endorsement

Arguments in favor of SF policy (and arguments against FCFS policy) focused on the policy being medically and/or ethically “right.” For example, participants discussed how SF policy makes medical sense: it is simply triage.

*Participant-102: Having been a medic, working in the medical field, I always go for the triage thing. It’s just automatic.*

Participants pointed out that it is important to get treatment to those who need it most.

*Participant-106: If you’re sicker you treat them first. It’s the same way if you got two women in labor, who are you going to help first? The one that’s more dilated and the one that’s labor pains are coming faster, you know? It’s just simple. I don’t believe in that, period. No first come, first served. You help the people that needs the help the worst.*

Ensuring treatment for those who need it most is a more ethical approach than FCFS:

*Participant-108: The only problem with the first come, first served and being served, is if you serve those folks because they came first and now you have no medicine, and the worst person comes in, what do you tell him? “I’m sorry, we gave them to other people who just weren’t as bad as you but we gave it to them anyway because they came here first.” That is unethical...*

*Participant-111: One’s 90 years old and his liver’s sick and he’s got this and maybe a viral load; in this case, 60 years old, he’s got the same viral load. They’re both the same, who’s going to get it? [ … ]*

*Participant-107: So use your Hippocratic Oath, which one are you going to save?*

*Participant-110: Well, then I have the choice to make the longest quality of life.*

SF policy also appealed to Veterans’ willingness to help and make sacrifices for fellow Veterans.

*Participant-110: All military people have an essence of taking care of their own. If you’re in a battalion and this guy is hurt worse than you, you’re going to try to help him [ … ] I don't know but that’s kind of been inbred into me in the military, as a military person, I would just say that this sickest has to go first.*

*Participant-119: … if someone’s sicker than me, I’d rather them get the treatment, because I’m going to get it, but maybe for them going first, they’ll survive...*

#### Concerns about sickest first policy

Even though the SF policy made medical and ethical sense to our participants, when they began to think about how to implement the policy, they realized it would not be easy. They pointed out that the SF policy requires a definition of “sickest” and should include specification of any exceptions for severe disease or risky behaviors.

*Participant-113: How would you come up with who is the sickest without some type of consult? How would you, let’s say me and you are both close together in our sickness but yours is just a little bit worse than mine? I mean, that’s where I’m stuck at right there.*

Participants also worried about how to decide when someone may be *too* sick to benefit from treatment.

*Participant-108: I would think that you’d have to think of the sickest person, how sick? Is he too sick for this to really, for us to put all this into him? Now should we go down to B Person, who’s as sick but not quite as sick?*

*Participant-111: So the con is that you have to have a sophisticated system...*

*Participant-108: You should have a number, a number that you look at to decide who’s the sickest.*

*Participant-123: … the con of it is, is sickest first sometimes people go through it and they’re too sick for it to really help them.*

*Participant-127: Depending on the stage.*

*Participant-123: I mean they need the treatment to help them with their liver condition but they probably, the system is so broke down, by the time you get that sick with it, then it’s just not going to help them. It’s going to prolong one, but you’re damaging the others.*

Another concern about the SF policy revolved around the need to balance how sick a Veteran is with their continued engagement in risky behaviors. If the policy prioritizes sicker Veterans, with no regard for their risk behaviors or adherence, other Veterans will have to wait, potentially putting them at risk.

*Participant-115: What about somebody who has 60% damage, I have 5% less but they drink all the time? Do they deserve treatment first because they’re sicker?*

### Toward better care: reducing barriers and improving policy

In both small group sessions, we asked Veterans to suggest ways to make care better. In the first session, we asked for ways to reduce barriers to care; in the second session, we asked how policies for prioritizing access could be improved to make them more effective and fairer. Their advice addressed weaknesses in the proposed policies and the barriers to care they identified, offering a roadmap for improving access to the limited resource of CHC treatment. Their advice focused on: a) fine tuning the assessment of health status, b) taking into account behavior and lifestyle, c) improving education and support, d) improving access and reducing costs, and e) promoting more just decision making.

#### Health status

The SF policy would require a consistent and fair approach to assessing health status. Recognizing this, participants suggested modifications that took into consideration factors such as age, disease severity, and overall health of Veterans with CHC.

*Participant-109: … so you’re going to waste the treatment on the people that aren’t even going to make it and let the people that would benefit, not get the treatment?*

*Participant-111: I agree with you, in triage in Vietnam, you had a belly and lung wound, we’re skipping you and we’re going for the amputation. Facilitator: So you’re not looking for sickest, you’re looking for...*

*Participant-111: For survivability.*

*Participant-109: Sickest first period won’t work, it just won’t work.*

*Participant-111: We had to do that triage in Vietnam.*

*Participant-110: That’s where it comes down to overall health and you have to have some criteria for that.*

Age, as a proxy for health status and life expectancy, was also discussed as a factor to take into consideration.

*Participant-101: Should there be an age limit? [...] This is my question to the group, should there be an age?*

*Participant-102: Well, looking at, you have to think about age, what other issues do they have and it’s better to ask the patient, “Do you think this is helping you, because you have these other issues, do you think this is going to help you in the long run?”*

The topic was a sensitive one, with disagreement about denying help because of (old) age.

*Participant-106: ...absolutely treat the younger person if they’re equally sick.*

*Participant-104: What if it’s not even younger but rather who would have a better quality of life?*

*Participant-102: … you can’t really look at age too much anymore.*

#### Behavior and lifestyle

In theory, “sickest first” sounds ideal, but what if the sick Veteran behaves in ways or has a lifestyle that reduces the value of the treatment? Many participants felt behavior and lifestyle should be taken into account.

*Participant-111: ...if the patient is intending to continue the risky behavior that caused this, if they have no intention of quitting their IV drug use or quitting the behavior that caused what they have […] It’s like why would I choose you even though you’re sickest, because you are not even mentally saying you’re going to do anything with that medication?*

Some participants also suggested pre-screening to assess merit and/or potential treatment adherence.

*Participant-120: … behavior kind of like lets you know what’s up, if they’re really serious.*

*Participant-126: What they’ve done and what their plans are to do, it should be put in the medical records. It shouldn’t just come from a first-time glance, it should be an actual process.*

*Participant-125: I think you should go before like a psychiatrist or a therapist or something that explains everything and see if you fit that criteria, because if your lifestyle’s not going to change, you’re just wasting everybody’s time and money.*

*Participant-115: I have to have some kind of questionnaire. [...] I think instead of going with sickest maybe you should go by who’s more deserving, the guy who’s going to take care of his liver or the guy who doesn’t really care, having a good time is more important.*

*Participant-126: Yeah, there’s got to be a weed-out process.*

*Participant-123: We’re making sure they’re not going to blow that money. [...]*

*Participant-126: Who are we weeding out? The people that don’t really want to complete the program.*

*Participant-120: There’s got to be some kind of a screening.*

However, a few disagreed or had concerns about taking into account Veteran behavior and lifestyle.

*Participant-106: There’s no perfect person on the earth. You’re doing something, you’re gluttoning, you’re a gambler, you’re eating, you’re drinking, you doing something. I don’t care who you are, you’re not perfect, right? But you’re still on the humanity side. You treat the sick person, it’s simple.*

*Participant-126: … we have to give everyone the benefit of the doubt, you have to from the beginning but whatever their history, whatever they do from here on out, it should lead to where they get continued help.*

#### Education and support

Participants also discussed ways to help patients overcome knowledge and other barriers that may keep them from completing treatment.

Participants saw the media – both old (TV) and new (social) – as a way to promote access by increasing knowledge and resisting stigmatizing images of hepatitis C.

*Participant-110: ...maybe do a television advertising outreach program asking all Veterans, symptoms or no, to please come to a VA facility in their area and get a hepatitis C test or go to their own doctor if they don’t want to come to the VA, and say you can have it and not know it and not have any symptoms, please, you know just please everyone go get tested for your own good.*

*Participant-101: ...we need, like there’s a Facebook out there, how about a VA book, how about a place to go where I can, "Hey, I’ve got hepatitis C …*

Some participants proposed CHC classes for Veterans.

*Participant-116: Well, they have classes for weight loss, they have classes for smoking, so why don’t they have it, since hepatitis C is supposed to be a big issue, have classes on that?*

*Participant-113: I agree, because you know like education is important. When you know better, you do better.*

Others proposed counseling and peer support.

*Participant-121: Counseling, I believe that we do need counseling, and not only just for us, but for our family to understand this disease …*

*Participant-127: A mentoring program [...] Well, like somebody that’s been through the program and says, “You know, we’re going to do it this way. I’ll work with you. If you need help, give me a call.”*

#### Improving access, reducing costs

Participants also discussed different ways to increase access to testing and treatment, as well as reduce the costs of providing treatment to patients with CHC.

Some recommended providing access to universal testing.

*Participant-107: I think the blood test for every new patient would be the easiest way to screen them out for any of their health problems.*

*Participant-111: Right, both at the end of the Army and when they enter the VA.*

*Participant-110: I agree with you, I think when you enter the VA healthcare system, the first thing they usually do is give you a CBC, a broad-spectrum blood test and I think the hepatitis C test should be included.*

Others focused on the need for transportation.

*Participant-123: … if they had transportation available for those people that were going to go through the hepatitis C program …*

*Participant-106: You have yellow cabs, why not have Veteran cabs?*

Some participants wanted to reduce costs (and increase access) by targeting pharmaceutical company profits.

*Participant-115: The government regulates everything else, why don’t they regulate something like this? Because the pharmaceutical companies are so rich they have bought the politicians.*

*Participant-126: Maybe it’s [time] for our policymakers to finally stand up to the pharmaceutical companies to get the price even lower because this is just a price that the pharmaceutical companies set.*

#### Better decision making

Participants also suggested creative ways to improve how allocation decisions are made.

One group suggested a “point system” to take into account different Veteran circumstances.

*Participant-110: For every criteria you come up with, you come up with another criteria to modify that criteria because it’s like what’s sickest but you’ve got to consider the overall liver health or the age and lifestyle, their age, does it really matter?*

*Participant-111: That’s why, you have to give these, I mean one way some clinics do is to give point values to all these and the highest point guy goes through.*

One participant suggested a committee of doctors should decide.

*Participant-112: If you add a modification then you would add a panel of doctors to determine who’s the sickest first. [...] after you got a group of folks that you was ready to treat, then you have a group of professionals, doctors to actually determine who is the sickest.*

Another participant was content to allow a doctor to make the decision.

*Participant-114: Again, I think that the doctor, professional people know about the sickest, the degree of sickness, I mean you know because I don’t know, I’m not a doctor and I don’t know anything like that, so I think that’s really in the doctor’s ballpark.*

At the end of the day, participants recognized the difficulty of deciding who gets treated first.

*Participant-110: ...a group of people is setting criteria for how to decide who gets it and when they get it and why they get it according to this criteria and we have just found out that it is not easy and that we cannot even come up with something that’s like 1–2-3 step.*

## Discussion

Patient voices are often overlooked in policy discussions, despite evidence suggesting that patient engagement can promote successful implementation by leading to better outcomes, improved patient satisfaction, and lower costs [20]. Our study demonstrates the feasibility and the value of using DD to meaningfully involve patients in conversations about health policies that are complex and require the balancing of competing priorities.

Our DD session offers an example of how to generate substantive patient engagement in order to inform the creation and implementation of health policy. Careful planning, review of the educational materials, the use of trained facilitators, and the availability of experts to answer questions about the policy/issue being considered combine to prepare and facilitate high level conversations among participants [15]. In this study, as in other deliberative exercises [14, 21, 22], participants reported high levels of satisfaction, saying they were listened to, respected, and that the process was fair.

Our primary aim was to assess of the value of deliberative techniques for eliciting Veteran preferences for the allocation of CHC treatment, an expensive (and hence, scarce) treatment resource. Our results show that DD is not just a way to promote “buy-in” to new health policies, but a method that can uncover creative and useful suggestions for designing and implementing those policies. In an era that values shared decision making in the clinic [23], DD is a way of extending that idea to allow patients to collaborate in policies that govern clinical care.

Pharmaceutical companies will continue to develop effective but expensive treatments for other diseases, forcing policymakers, payors, and healthcare systems – including state and federal programs – to find novel and pragmatic ways to provide these costly medications to patients. Our work offers a model for soliciting patient preferences, allowing their knowledge of the barriers to care and their creative and novel program ideas to be used to craft policies for the fair and acceptable allocation of scarce medical resources.

Our research has limitations. Given limited time and concerns about cognitive overload, we could not thoroughly educate participants on every issue of importance to this topic, such as the wide range of VA expenditures that compete with CHC treatments for limited financial resources or the role of dependency disorders and other conditions in the spread of CHC in the Veteran population. As such, we chose to focus the education on the key medical details of CHC and its treatment, as well as the overall issue of resource scarcity. Additionally, this was designed as a small-scale, proof of concept study, and while we are able to recruit a diverse sample, we do not have sufficient numbers of participants to draw meaningful conclusions regarding differences between subgroups of participants, such as those that have and have not been diagnosed with hepatitis-C. Further research with a larger sample will allow a broader assessment of Veteran preferences regarding the allocation of scarce medical resources. The results of those studies, combined with what have learned, can be used, not only to shape policy, but to inform the content of future educational materials. Comparative research, done with members of other healthcare systems, should also be done to learn how the opinions and suggestions of those patients are like and unlike those of Veterans. It is important to note that the VHA is one of the few healthcare systems that has successfully treated a majority of CHC patients.

## Conclusions

Democratic deliberation methods proved to be effective in eliciting patient insights and preferences for healthcare policies. In this study we learned that Veterans favored a modified SF policy for addressing the allocation of scarce healthcare resources for CHC treatment. Furthermore, our participants provided a variety of thoughtful ideas for how to overcome barriers to access and improve quality of care. Incorporation of the preferences and insights of patients is critical for optimizing care when implementing healthcare policy decisions for resource-limited environments, both within the VHA and in other healthcare systems.

## Supplementary information

**Additional file 1 **Pre-Post Deliberation Comparison (*n* = 30). Comparison of survey data from before and after the deliberation.

**Additional file 2.** Thematic Domains Discussed by Small Groups. Table of additional excerpts used in the thematic analysis.

**Additional file 3.** Semi-Structured Interview Guide. For preliminary interviews conducted to inform the design of deliberation materials.

**Additional file 4.** Baseline Survey. Administered at the beginning of the day, before the start of the deliberation.

**Additional file 5.** Follow-up Survey. Administered at the end of the day, after the deliberation.

**Additional file 6.** Facilitator Discussion Packet. Given to the small group facilitators to help them guide the discussions.

**Additional file 7.** Participant Discussion Packet. Given to participants to help them follow the discussions.

## Data Availability

The full dataset is not publicly available due to confidentiality policies. Some additional data may be made available upon reasonable request.

## Abbreviations

CHC: Chronic Hepatitis C
DD: Deliberative Democracy/Democratic Deliberation
FCFS: “First Come, First Served” policy for treating Veterans with CHC
HCV: Hepatitis C Virus
SF: “Sickest First” policy for treating Veterans with CHC
VAAAHS: VA Ann Arbor Healthcare System
VHA: Veterans Health Administration
