# Supplementary material for: Eliciting patient views on the allocation of limited healthcare resources: a deliberation on hepatitis C treatment in the Veterans Health Administration
Source: BMC Health Serv Res. 2020 May 1;20:369. doi: 10.1186/s12913-020-05211-8 (PMC7193376; doi:10.1186/s12913-020-05211-8)
Supplement: Supplementary file 1 — Additional file 1 Pre-Post Deliberation Comparison (n = 30). Comparison of survey data from before and after the deliberation. [file 12913_2020_5211_MOESM1_ESM.docx]

# Additional File 1: Pre-Post Deliberation Comparison (n = 30)

|  | **Baseline Survey**  **n (%)*** | **Follow-up Survey**  **n (%)*** |
| --- | --- | --- |
| **When there are limited resources, which of these policies for treating Veterans with Hepatitis C *should* the VA adopt?** |  |  |
| **First come, first served** – Veterans are treated in the order in which they come in for treatment. | 4 (14) | 2 (7) |
| **Sickest first** – Veterans are treated in the order of how sick they are from Hepatitis C (e.g. symptoms or complications such as liver cancer or cirrhosis). | 24 (86) | 25 (93) |
| On a scale of 1 (Not strongly at all) to 10 (Very strongly), how strongly do you feel about the option that you chose in question 2? Mean (SD) | 8.5 (2.1) | 8.8 (1.4) |
| When there are limited resources, which Veterans with Hepatitis C should be treated *first*? (choose one) |  |  |
| Veterans who have liver cancer. | 6 (21) | 2 (7) |
| Veterans who have liver cirrhosis but do *not* have liver cancer. | 1 (4) | 4 (14) |
| Veterans who have symptoms, but do *not* have liver cirrhosis or liver cancer. | 3 (11) | 5 (17) |
| Veterans who do not have symptoms. | 0 (0) | 0 (0) |
| *All* Veterans should be treated without delay *regardless* of symptoms or whether they have cirrhosis or cancer. | 18 (64) | 18 (62) |
| Satisfaction with care at VA facility  (from Very Dissatisfied, 1, to Very Satisfied, 5), Mean(SD) | 4.3 (0.8) | 4.5 (0.7) |
| “I trust the VA Health Care System to decide who gets treated *first* for Hepatitis C.”, Mean (SD) † | 3.7 (0.9) | 3.5 (0.9) |
| Overall trust in *VA Healthcare System* (Score ranging from 0 to 100, with higher numbers indicating more trust), Mean (SD) † ‡ | 66 (17) | 69 (16) |
| “I trust VA *health care providers* (e.g. primary care doctor, liver doctor) to decide who gets treated first for Hepatitis C”, Mean (SD) † | 3.7 (0.9) | 3.7 (0.8) |
| Overall trust in *VA Primary Care Team* (Score ranging from 0 to 100, with higher numbers indicating more trust), Mean (SD) † § | 63 (18) | 62 (14) |
| Number of correct knowledge questions out of 7, Mean(SD) \|\| | 5.4 (1.6) | 6.4 (0.6) |

* Percentages sum vertically. Valid percentages of non-missing data are shown.

† Response options range from Strongly Disagree (1) to Strongly Agree (5).

‡ This scale was adapted from the 9-item Revised Health Care System Distrust Scale: <https://www.ncbi.nlm.nih.gov/pmc/articles/PMC2517896/>
(e.g., specifying the “*VA* Healthcare System” rather than “Healthcare System”).
To allow for direct comparison with the Trust in Physician Scale, these scores were also transformed to 0-100 and reversed so that higher numbers indicate more trust.

§ This scale was adapted from the 11-item Trust in Physician Scale: <https://www.ncbi.nlm.nih.gov/pubmed/12579593>
(e.g., specifying the “VA Primary care team” rather than “my doctor”).

|| This knowledge/comprehension test contained 7 True/False questions assessing participants’ understanding of Hepatitis C both before and after the expert presentations/deliberations.
