# Supplementary material for: Eliciting patient views on the allocation of limited healthcare resources: a deliberation on hepatitis C treatment in the Veterans Health Administration
Source: BMC Health Serv Res. 2020 May 1;20:369. doi: 10.1186/s12913-020-05211-8 (PMC7193376; doi:10.1186/s12913-020-05211-8)
Supplement: Supplementary file 2 — Additional file 2. Thematic Domains Discussed by Small Groups. Table of additional excerpts used in the thematic analysis. [file 12913_2020_5211_MOESM2_ESM.docx]

**Additional File 2. Thematic Domains Discussed by Small Groups**

| **Theme** | **Example Quotes** |
| --- | --- |
| **Barrier to Treatment**:  Lack of Knowledge | Participant-106: I was considering the mental aspect of veterans that don’t even know how to get theirself treated for Hepatitis C and all that.  Participant-109: I think one of the barriers is the veterans themselves like me, I never heard of Hep C and I didn’t ask for the treatment [...] Some guys don’t even know about it.  Participant-110: I’d say that people are not informed. They hear the horror stories of the side effects of the interferon treatment and unless they’re avid TV watchers to see the Harvoni commercial, they have no idea that today they can treat it with a pill.  Participant-114: I had to ask my doctor, “Well what are symptoms of Hepatitis C?” and they were very vague and I don’t believe I experienced any of them all the time that I had it, it’s like, what symptoms?  Participant-119: When I caught Hepatitis, I didn’t know I had it [...] all they did was quarantine me [...] I just didn’t get no more information about it.  Participant-112: …Basically, most of it is, actually is all awareness of hepatitis C. I think there’s [...] a lot of people that really doesn’t know the severity of hepatitis C...  Participant-112: Yeah, and you see that, that information isn’t out, see, because like we were saying, the ads for advertising stuff is mostly directed toward the baby boomers and the older generation and not letting the young generation know that because of the opioid crisis and all of that, and the high drug use and stuff will cause Hepatitis C too.  Participant-115: I pretty much think everyone knows about Hepatitis C, everyone knows it can kill you. So, the biggest education is putting out information to where someone who might suspect that they have Hepatitis can go to get tested.  Participant-117: Lack of knowledge, their knowledge [...] If he doesn’t know what Hep C is or the seriousness of it, the illness.  Participant-103: …I would think the fact that some people don’t experience any symptoms would be a barrier, so they don’t even know that they have it. |
| **Barrier to Treatment**: Testing and Treatment Adherence | Participant-118: … they’ve wasted this medication that could have helped someone that was more willing to do what they were supposed to do because if this medication is so high and somebody’s just kind of playing around, they’ve wasted it for a veteran that could’ve used it.  Participant-109: Yeah, it's easier to be in denial.  Participant-109: … some people just don’t even like taking pills, although the veteran himself I believe can be a, in my case, the veteran himself was the biggest barrier all the way down.  Participant-109: … some people don't even like medicine. Some guys you can't get them to do anything.  Participant-110: Some people say, “I'd rather not know if I have lung cancer, I’ll just keep smoking cigarettes, when it happens, it happens. I don’t want to go find out,” I think that a lot of the people saying [...] If somebody said yes, it’s all, “We’ll test you for free, just come in and say you want the Hepatitis C test, it's one blood test, just get the test.” [...] If you don’t care, no, but I mean if you're into bad behaviors and you just want to continue those bad behaviors...  Participant-110: Some people are like, “I’m not, I’m not going. I don't even want to know. I don't want to know if I have this or not. I don't even care how easy it is to cure it. I don't care if I have it because what does that mean if I find out I have it?”  Participant-110: Don’t trust the VA at all because they think they got it [i.e., hepatitis C] from the Army.  Participant-116: Some people just don’t want to find out.  Participant-113: … because you didn’t feel the symptoms, you weren’t in no great urgency to do anything about it, you know? You didn’t feel no different, or at least you didn’t think you did. […] I guess I’m saying that because I didn’t feel it, because the consequences wasn’t so great of me having it, you know “I can put it off until tomorrow.” So, like that, procrastination, “Maybe tomorrow.”  Participant-121: ...it’s trying to stay focused on taking medication because you’ve got so many other factors in your life, that kind of like, “Oh, did I forget this?” and if I miss a dose, then do I add-on to it or you just call and let them know?  Participant-123: Well they start, but they make it bad for the people who really need the Hep C because you’ve got these people, they’ve started the program, they spent the money on the 28 pills that they give you every month, they spent that to get your regimen in, they bought your whole regimen, even though you're getting 28 pills a month, they bought your whole regimen, you start that thing off, but you don't complete it. There is money lost.  Participant-123: We had people dropping out. One guy dropped out. He didn't want to quit drinking. The other people dropped out because they didn't have transportation. We had one person dropped out, just refused to keep taking it on time, you know, you get up every morning and you take the pill.  Participant-125: I’ve talked to different veterans and stuff and a lot of them do not want to take Hep C treatment because they're afraid they're going to lose their disability because of it.  Participant-105: I guess that just brings to mind just an individual having a lack of trust about the treatment maybe because they have some side effects they don’t like or for any number of reasons. |
| **Barrier to Treatment**: Vulnerable Populations and Stigma | Participant-105: Well I was thinking of other medical complications that the patient may have like alcoholism and if they are still actively using alcohol that might affect treatment overall…  Participant-118: If you’re homeless you don’t have an address so you ain’t getting nothing…  Participant-101: Is shame a possible thing that we wouldn’t, you’d ignore your symptoms and wouldn’t talk to a doctor because you don’t want anybody knowing what your past… possibly?  Participant-102: I can see that or IV drug use, same thing. Participant-128: Stigma. Participant-101: Yeah, you want to hide, conceal, say, “So I’m not going to tell anybody, I’m going to die of hepatitis C and liver cirrhosis.”  Participant-110: ...they think that there’s some kind of stigma to being… a drug addict or they’re not sure how they got it, they don’t know that there’s a new treatment, you take 1 pill a day for a few months and you can be cured.  Participant-114: … before, you didn’t talk about Hepatitis C in groups or you didn’t talk about it to the next person because hey, a lot of them thought that it came from drug use...  Participant-121: ...a lot of people are scared to tell their doctors everything and then their doctors find out something else…  Participant-113: …if you’re just out there doing drugs and getting high and stuff like that, why would we want to spend that kind of money on you?  Participant-120: They don’t want to change their old habits, their bad habits, drugs, alcohol. You’ve got to let the party life go, there’s no party anymore when you’re treating for that, I’m serious.  Participant-117: Mental health [...] Just like my friend, you know, it’s hard to get him to go get the basic things. It’s hard to try, it’s hard to convince him.  Participant-115: ... if you’re homeless, you probably also don’t have access to health care, even getting a way to the VA. So, you may not know that you have Hep C and if you do find out you have it. Being homeless means you’re probably without transportation and no one’s exactly going to pick up a hitchhiker who’s homeless.  Participant-115: Somebody who shoots up drugs is going to have to first want to be able to get rid of the addiction or get clean and if he doesn’t want to get clean, then he’s not going to be interested in a trial program or whatever, getting help.  Participant-112: It’s a barrier, especially among young folk because they may not be drug users and stuff and have Hepatitis C and they don’t want to go to the doctor to have their self-checked out because they automatically think that oh, I’ve been using drugs, I’ve got Hepatitis C. |
| **Barrier to Treatment**: Transportation and Access | Participant-122: … it’s a policy that someone in Washington has decided, because it costs a lot less to allow 100,000 vets to die than to treat them.  Participant-122: So, by putting our lives on the line, I don't really accept someone telling me it costs too much. I think that's an insult […] I mean if a person needs to be tested for hepatitis C and it costs $10,000, you know what, this guy was going to die for us, for our nation.  Participant-102: … so depending on where the person’s from, how much is it going to cost to, say if they can’t drive themselves, how much is it going to cost to transport people from one place to the other?  Participant-128: ... I think some of our politicians now-a- days, if they don’t serve in any kind of a military or branch of service [...] and I don’t care whether it’s a Republican, Democrat or an Independent, I don’t care. If that person does not know what he, she or who went through during the era, they don’t understand the complications that some of us have now and they don’t understand just because this guy has got money and this guy does not, both of them should be treated the same because both of them served their time in the military.  Participant-101: … they’ve got 5 VA hospitals, 300 miles away from wherever you are, it’s not in your backyard, that’s tough. I couldn’t even imaging having to travel 3 hours or something to a hospital.  Participant-106: ... one of the top priorities is to get the veteran there so transportation should be more easier and prevalent for a veteran.  Participant-104: I’ve heard of certain medications the VA doesn’t allow or use because they can’t get the pharmacy companies to lower the price and afford it.  Participant-104: There’s a lot of barriers for veterans trying to get help and I think it’s a lot of politics and a lot of budget is always, always comes back to dollars and cents.  Participant-111: ...the idea that the VA’s going to test everybody for all the common diseases is nil. They don’t want to do that because they think it's going too expensive to treat, or they don’t think they have enough doctors, it’s all bullshit because this can all be done by a technician and a blood test and then only follow up on the sick people.  Participant-123: The VA used to give me transportation, […] but now as far as travel is concerned, you can't get travel now unless you're blind, you're missing a limb, or your wheelchair-bound.  Participant-123: That's another [barrier] we can put up there… government cuts with the VA. People went out there and served our country. We ought to be able to be transported back and forth to the hospitals and you know they're cutting money every time from the VA.  Participant-120: … there's not enough doctors on staff to treat. I've seen it in the VA.  Participant-126: [Referring to rural areas] They only have little clinics and they are way understaffed.  Participant-126: A lot of people don't have rides. That's just a simple thing.  Participant-118: … the doctors are already so overloaded that’s why you only get 15 minute sessions, so I think it would be difficult because of all the other like I said the barriers and things of hiring people, the cost, and the VA just doesn’t have time or money to chase down somebody who’s not going to make those efforts. |
| **SF vs. FCFS**: SF Policy Pros | Participant-101: ...until you have the cirrhosis or the cancer, you’re never going to die of this, you can live 30 years with it, so why concern treatment here when it takes 30 years for the cirrhosis to show up? [...] Save the guys that are hurting.  Participant-102: … the sickest I feel, those have got a bigger problem, need the most help first.  Participant-102: Having been a medic, working in the medical field, I always go for the triage thing. It’s just automatic.  Participant-103: I would say a pro is the fact that the person that needs it the most would get treated.  Participant-104: … I know myself that I would be willing if I knew I could make it to next year and somebody else couldn’t, I would say alright.  Participant-105: … the doctor takes a Hippocratic oath and wants to first do no harm. Iit would just on that basis, be really difficult to be looking at a patient who’s sick, very sick, have to say well, we have this first come, first served policy...  Participant-106: If you're sicker you treat them first. It’s the same way if you got two women in labor, who are you going to help first? The one that’s more dilated and the one that’s labor pains are coming faster, you know? It’s just simple. I don’t believe in that, period. No first come, first served. You help the people that needs the help the worst.  Participant-107: I’m thinking about the Hippocratic oath and regardless of your first come or first served, isn’t it the doctor’s obligation that if you are very sick, for them to treat you right away?  Participant-108: … it is the right thing to do. But because he is the sickest, he should be the first.  Participant-108: The only problem with the first come first served and being served, is if you serve those folks because they came first and now you have no medicine, and the worst person comes in, what do you tell him? “I’m sorry, we gave them to other people who just weren’t as bad as you but we gave it to them anyway because they came here first.” That is unethical...(  Participant-111: I don't know, I think, that the majority, a lot of veterans have good hearts and would say, Oh my God, look at him those yellow eyes and a great big belly, and I have to let you go first.”  Participant-110: … a con of first come, first served is that I could come first and have no symptoms and the person behind me could be extremely sick and then I would get treated before that person, that would be a con of first come, first served. Participant-107: Yeah, because it’s like the medical staff is obligated to do the right thing.  Participant-110: All military people have an essence of taking care of their own. If you’re in a battalion and this guy is hurt worse than you, you’re going to try to help him and when you get to the medics, […] you want them to take care of him first and you can hold up. I don't know but that’s kind of been inbred into me in the military, as a military person, I would just say that this sickest has to go first.  Participant-114: In the ER, Emergency Room, people come in sick all the time, but the sickest one or the ones that have the most serious injuries are treated first. I don’t even know, yeah triage...  Participant-119: … if someone’s sicker than me, I’d rather them get the treatment, because I’m going to get it, but maybe for them going first, they’ll survive...  Participant-117: Sickest first and that’s it. [...] And then that’s a oath that a doctor has to take. |
| **SF vs. FCFS**: SF Policy Cons | Participant-123: … the con of it is, is sickest first sometimes people go through it and they’re too sick for it to really help them. Participant-127: Depending on the stage. Participant-123: I mean they need the treatment to help them with their liver condition but they probably, the system is so broke down, by the time you get that sick with it, then it’s just not going to help them. It’s going to prolong one, but you’re damaging the others.  Participant-102: Sickest first? They don’t know how sick they are unless they do a biopsy, then that’s more money that they could use for like testing everyone. [...] Participant-101: Yeah, they said it’s very difficult when they, how sick they are, they don’t know.  Participant-104: I mean say you had to, is there a level you have to reach to be determined that you fall into the category to be decided if you're sickest first?  Participant-118: Why wait until you get cirrhosis before they treat you?  Participant-103: Well, I mean it sounds good when you just say it, but suppose all of us are sick, […] but suppose I’m the most sick so I get treated first and suppose I don’t take my medicine at all [...]and when you come back the next month and I’m still the most sick and I keep coming back and all of you die because you waiting on me to be treated first.  Participant-103: I had one more comment about the sickest first and I would say it was a con. The fact of someone that continually abuses their selves or neglects their medication or things of that nature, they would get treated first even if they were worse and they’re not even trying to get healthier. So if they still doing high risk things or not taking the treatment or not attending appointments they still keep getting treated before a person that’s steadily getting worse and worse and worse that’s trying to get treatment.  Participant-113: How would you come up with who is the sickest without some type of consult? How would you, let’s say me and you are both close together in our sickness but yours is just a little bit worse than mine? I mean, that’s where I’m stuck at right there.  Participant-113: … this group [of waiting Vets] will be neglected, totally neglected, because by the time you got back to them that ‘First Come, First Served’ list, somebody sicker would come in again.  Participant-115: What about somebody who has 60% damage, I have 5% loss but they drink all the time? Do they deserve treatment first because they’re sicker?  Participant-120: Yeah, but what if you get someone in there that’s got a severe problem as far as the Hep C but they’re not quite as serious about getting treatment?  Participant-108: I would think that you’d have to think of the sickest person, how sick? Is he too sick for this to really, for us to put all this into him? Now should we go down to B Person, who’s as sick but not quite as sick? Participant-111: So the con is that you have to have a sophisticated system... Participant-108: You should have a number, a number that you look at to decide who’s the sickest. |
| **Policy Improvement:**  Health status factors | Participant-116: If you’ve got cancer and someone else don’t have cancer, why not treat that person who don’t have cancer first, although that person who has cancer is the sickest?  Participant-122: … is it end-stage? Is the rest of his body being affected by it? Do they have cancer now, liver cancer? So that’s, and if they’re at end stage, they can’t do anything for them. If they’re at beginning stage, there are a lot of options.  Participant-101: Should there be an age limit? [...] This is my question to the group, should there be an age? Participant-102: Well, looking at, you have to think about age, what other issues do they have and it’s better to ask the patient, “Do you think this is helping you, because you have these other issues, do you think this is going to help you in the long run?”  Participant-101: I think we shouldn’t treat terminal anything, cancer, cirrhosis.  Participant-106: I think your age and stuff like that should come into in consideration.  Participant-106: ...absolutely treat the younger person if they’re equally sick. Participant-104: What if it’s not even younger but rather who would have a better quality of life?  Participant-104: So you have a 90% chance of survival, you have a 80% chance of survival so we’re going to give it to the 90% person.  Participant-104: As long as the budget’s available we’re going to keep an option of a very sick person coming in and getting taken care of right away.  Participant-104: Okay, so therefore, we could make our policy, our policy could be that we deal with first come, first served but if we still have budget left and somebody real sick comes in then they get taken care of.  Participant-102: You’re looking at comorbid health issues. If you have cancer, if you have diabetes, if you have a heart problem, stuff like that […] they have to look at all the other health issues that you might have and the other medications you’re taking.  Participant-102: …you can’t really look at age too much anymore.  Participant-103: For severe cases, you let them go ahead.  Participant-107: … I say the sickest people clause comes in through initial first come, first served screening, whoever pans out to be really sick, gets in line first.  Participant-108: Well, you want to consider the length and quality of life and all that...  Participant-109: … so you’re going to waste the treatment on the people that aren’t even going to make it and let the people that would benefit, not get the treatment. Participant-111: I agree with you, in triage in Vietnam, you had a belly and lung wound, we’re skipping you and we’re going for the amputation. Facilitator: So you’re not looking for sickest, you’re looking for... Participant-111: For survivability. Participant-109: Sickest first period won’t work, it just won’t work. Participant-111: We had to do that triage in Vietnam. Participant-110: That’s where it comes down to overall health and you have to have some criteria for that.  Participant-110: I say another modification would be the state of health of the rest of your body...  Participant-118: Yeah, if you only have three months live, I mean, then it would be a waste of any kind of medication.  Participant-118: Do sickest first, I agree. You should take age into consideration, you should take other health factors into consideration. I mean if I’ve got so many other diseases I know I got six months why should I step in and take that treatment, okay I might be sicker than everybody else but, not to be crude but it’s going to be a waste of funds...  Participant-111: One’s 90 years old and his liver’s sick and he’s got this and maybe a viral load; in this case, 60 years old, he’s got the same viral load. They’re both the same, who’s going to get it? […] Participant-107: So use your Hippocratic Oath, which one are you going to save? Participant-110: Well, then I have the choice to make the longest quality of life.  Participant-119: I would say age factor. A younger person probably could have a little bit more time then, you know, because they’re probably more active in their lifestyle then someone that’s older than, you know what I’m saying, or as their health-wise, not as active as a younger person. Facilitator: So, you are saying that younger folks can wait? Participant-119: Exactly, you know, can consider it a little bit, “I might have to wait another thirty days. This person is a little bit older than me.”  Participant-119: …when they said about the liver, that’s irreversible and that person got that cirrhosis, it can’t be cured, but you want to stop, you know, it’s like if I got cirrhosis and you didn’t, I think you should be served so you won’t get in the state where I’m at, you see what I’m saying? Yeah, because that’s a, in other words, I’m incurable. You can be cured, but I can’t.  Participant-120: Alright, now that scale of sickness, okay, let’s say hypothetically that person with the Hep C virus is really at the peak of it as far as, the scale of, the level of severity of the disease. Let’s say he’s skyrocketing high, then what? The scale of sickness? […] That person moves to the top of the list for treatment... |
| **Policy Improvement:**  Behavior and lifestyle factors | Participant-122: … if he’s going to his appointments on time, he’s making all these appointments, he’s taking his medication, he’s trying, he’s consulting the doctor, you, the doctor or the system would look at this patient and say, this veteran patient, say okay, he’s doing everything, following every point that we told him to, so we’re going to make sure he’s taken care of. Now you get another veteran who doesn’t show up, getting drunk, getting high, doesn’t like to take his medication, doesn’t give a shit about anything. What are you going to do? You’re trying to treat him but he doesn’t want to, he’s still doing drugs, so now you’ve got what I call the patient specific treatment, okay. One of them is giving it 110%, the other one is giving it negative 10%. Who do you treat? Who do you invest the 90,000 or the 10,000 in? […] So you’ve got two different patients and you look at them, you say okay, we can treat this guy, he wants to be cured. This guy really doesn’t care, he’s still shooting up drugs.  Participant-102: We could run them both together when you do like the specific triage system, that kind of breaks it down a little bit more as far as, “Okay, who’s coming to their appointment?” Like the newly diagnosed people, you can weed out very quickly by just looking at records whether a person is compliant or not with treatment.  Participant-105: ... if a patient is abusing alcohol I think the patient has to be an advocate for their own health […] I don’t like the idea of depriving anyone of care, but if someone is making the effort themselves to try to get well then that should speak for something.  Participant-118: I spent all my life partying and stuff. Why should I get to go first when somebody else is taking good care of themselves?  Participant-118: …if there’s somebody that’s you see in the past that cancels a lot of appointments, don’t show up for appointments, the doctor hands you a prescription but, six months later, you're calling in for a refill well, you should’ve had it two months ago...  Participant-111: ...quality of life of those years is also important, like are you going to live out your life as an alcoholic, you can live out your life as a concert pianist or a cardiac surgeon. Participant-110: Lifestyle. [...] The lifestyle has to figure into it.  Participant-111: ...we have to know, of these patients who really wants to get treated and who’s going to cooperate, that’s one of the first questions to find out.  Participant-111: ...if the patient is intending to continue the risky behavior that caused this, if they have no intention of quitting their IV drug use or quitting the behavior that caused what they have, if you talk to them, if they had some psychological counseling and they say, “Eh, I’m only here to get my hepatitis C cured. I don’t care about not being a heroin addict, right, I’m going to continue doing that.” Okay? It’s like why would I choose you even though you’re sickest, because you are not even mentally saying you’re going to do anything with that medication.  Participant-119: … the person’s lifestyle, if they’re still out there drinking, not taking it seriously. I mean, somebody’s sicker than them that’s taking it seriously, should go ahead of them.  Participant-121: … regardless of what their lifestyle may be, even if they’re not taking their meds and they still come in, they still need to be treated, even though they’re not doing what the doctor had prescribed for them to do, but they still need to be seen if they’re at the sickest point in their lives, at their sickest point because you can’t make, you have to have sound judgement about this, about anybody that comes in as sick as this....  Participant-114: I don’t think a doctor, they don’t judge, oh yes they do, they judge on lifestyle […] but I don’t think it’s right.  Participant-123: … why, if I’m a doctor, why am I going to put you in a program and I know you’re going to go back to drinking and drugging? Why am I going to have the government spend $50,000 on you and you’re going to go through this program for 12 weeks, you’re going to go back to using and drinking? Hep C is going to come back on you, or you’re going to have your liver going to die. So, why am I going to spend $50,000 to get you that treatment?  Participant-123: ...if they're homeless you can actually screen them and you can find out, “If I was able to help you get over Hep C, would you come to the program? If I can get you in a room for the 12 weeks, would you stay and complete the program?”  Participant-115: I have to have some kind of questionnaire. [...] Well it’s like I said earlier I only have 5% damage, he’s got 40 but he goes out on Friday and Saturday night and gets trashed, I stay at home raising four girls and work every day. I think instead of going with sickest maybe you should go by who’s more deserving, the guy who’s going to take care of his liver or the guy who doesn’t really care, having a good time is more important.  Participant-115: What I’m saying is they need a way to verify a person’s lifestyle.  Participant-127: But if it’s to your caregiver, or your Primary Care giver to give you that referral, why would they give you that referral knowing your history and that you’re going to fail the program? That’s $50,000! I mean if I had $50,000 in my pocket I just wanted to throw away, it sure wouldn’t be on no drug treatment for $50,000, but they’re spending $50,000 per vet to help you with your life and if you don’t have enough respect of yourself, the dignity of yourself to want to improve your life to get something that works...  Participant-126: … you’re still handing to somebody that is homeless that don’t have a proper way to keep $50,000 [in medications] enclosed.  Participant-126: ...I think they should allow you to do the learning period and then give you the chance in between to change your life and your lifestyle choices and then go for your treatment.  Participant-126: … the doctors should say that it is a, works hand in hand, that the drugs and the alcohol is...you shouldn’t be allowed to do them if you get the medicine.  Participant-126: Yeah, there’s got to be a weed-out process. Participant-123: We’re making sure they’re not going to blow that money. [...] Participant-126: Who are we weeding out? The people that don’t really want to complete the program. Participant-120: There’s got to be some kind of a screening.  Participant-126: … we have to give everyone the benefit of the doubt, you have to from the beginning but whatever their history, whatever they do from here on out, it should lead to where they get continued help.  Participant-120: …find out who’s serious about the treatment and who’s not. Do it by a background check in their medical records to find out if there’s drug abuse, alcohol abuse.  Participant-120: I think regardless of how sick they are, they all get screened. Participant-126: Okay on that, just 10 years ago I wasn’t the same person I am today and if somebody judged me off my medical records 10 years ago, I wouldn’t probably get any help. So, I’m sitting here now that I’m a different person and trying to say that there needs to be a screening process [...] but now if you were to look, I’ve taken the 10 years and I’ve never missed a doctor’s appointment, I have everything, so I have a complete straight history. [...] But if I would’ve got sick right after I had come in for all my substance and all my other stuff? I would’ve been automatically disqualified.  Participant-120: … behavior kind of like lets you know what’s up, if they’re really serious Participant-126: What they’ve done and what their plans are to do, it should be put in the medical records. It shouldn’t just come from a first-time glance, it should be an actual process. Participant-125: I think you should go before like a psychiatrist or a therapist or something that explains everything and see if you fit that criteria, because if your lifestyle’s not going to change, you’re just wasting everybody’s time and money.  Participant-106: I mean everybody’s got a quirk. There’s no perfect person on the earth. You're doing something, you're gluttoning, you’re a gambler, you're eating, you're drinking, you doing something. I don’t care who you are, you’re not perfect, right? But you're still on the humanity side. You treat the sick person, it’s simple. |
| **Policy Improvement:**  Education and Support | Participant-104: ... one of the reasons I came here was to learn more about Hepatitis and discuss things about the treatment that vets get and there’s a way to overcome by teaching and letting people even know what it’s about […] All these years I haven’t known as much as I know now about Hepatitis.  Participant-103: Or they could come up with like some type of agreement or consent form, like have the family member or somebody take responsibility that they will make sure that you get this medicine. [...] I know like when you have like outpatient surgery or whatever or something and you can’t drive yourself home, you have an agreement that somebody signs saying that they will make sure that you’ll get home, etcetera, before they even do the procedure, something similar to that where they would say, “I’ll be sure this person takes his medicine for the next three months” or etcetera.  Participant-103: I think all of that, the not knowing where to go to get tested, the lack of symptoms and other medical issues all falls under the training and the doctors talking to you more and making brochures or something so everyone had access to the information. Maybe, they’ll understand more.  Participant-110: … giving them knowledge that they have the right to go to VA, that they should go to Veterans Affairs, that all of these systems are available for their use because they don’t tell you…  Participant-110: ...maybe do a television advertising outreach program asking all Veterans, symptoms or no, to please come to a VA facility in their area and get a hepatitis C test or go to their own doctor if they don’t want to come to the VA, and say you can have it and not know it and not have any symptoms, please, you know just please everyone go get tested for your own good.  Participant-109: Well, the VA does a pretty good job now of putting stuff on TV, pamphlets, stuff like that, mostly TV public service announcements and stuff, but the more the better, because it was getting past me.  Participant-116: Well, the TV commercials are helping that out on TV. […] they’re telling everybody that you know, how simple it is to get Hepatitis C, and they’re not using the drug use, and then the people that they do show, they look professional. I guess anybody can get it.  Participant-116: Well, they have classes for weight loss, they have classes for smoking, so why don’t they have it, since hepatitis C is supposed to be a big issue, have classes on that? Participant-113: I agree, because you know like education is important. When you know better, you do better.  Participant-118: … someone in the VA system that could just call and say, “Hey, have you taken your meds this morning? Why don’t you take it?” And hopefully that might work. I mean if it doesn’t, there’s another failed thing that at least the VA tried.  Participant-119: Talking to more veterans and having open, good communication with other veterans other than doctors which would pick, you know, go through this, be, “Hey, they made it through this, so look, I can do this too.”  Participant-121: With the TV commercial, everybody can see, they’re visualizing it, like, “Oh, just because I have Hepatitis C, doesn’t mean I don’t enjoy my life.” Participant-113: Yeah, more people start talking about it, people start telling you that would have never told you before like I found out another member of my family had it, he told me, “You know man, I’ve got that Hepatitis C,” and then the way he said it, I said, “Man, I’ve got Hepatitis C too,” So people, they don’t feel as stigmatized...  Participant-121: Counseling, I believe that we do need counseling, and not only just for us, but for our family to understand this disease as far as how it’s affecting our relationship with our spouses, our children, anybody that’s in the house, now that we’re having more extended families living together, and so they need to be aware and understand about this disease and how it’s affecting their relationship and communication with one another.  Participant-114: I think education would be a cure […] Yeah, a solution, because you can make an informed decision, you know? I don’t want to make a decision until know as much as I can about it.  Participant-120: ...mentoring is awesome. I like that, do you know what I'm saying? If you can find some, getting people involved that have experienced it before that’s gone through homelessness that have been there where these people are that you're trying to pull in and drag in, mentoring is important. It's just like having a sponsor around Narcotics Anonymous…  Participant-123: ...before we started our medications, we had a session we had to go to, to learn about Hep C and what treatments were going to be and they were trying to explain to us what different medications were, what side affects you might have. We had to go through this, I had to go one day a week for 4 weeks before I started the pills for more information.  Participant-126: I mean you walk through the door and if you have Hep C, they have all the pamphlets, they have all the stuff right there and that’s where I’ve always found out a lot of my information, but [...] you should be a little bit more visual, you know what I’m saying? People to say, “Hey, if you need information on this, then here’s where to come and get it.”  Participant-127: A mentoring program [...] Well, like somebody that’s been through the program and says, “You know, we’re going to do it this way. I’ll work with you. If you need help, give me a call.”  Participant-105: Well I mean I guess the obvious thing may be to have, you know, when people need a CPAP machine they go to these classes where they talk about all the specifics related to that or diabetes, same thing so some kind of class where you’re taught how important it is to take the medication and how long and that’s the only way that cures could happen.  Participant-112: Ways to overcome it? [...] Education [...] Public announcements, I mean.  Participant-112: The easiest to overcome is awareness, because I think that because of all the social media stuff that’s out there, you know what I’m saying, you can get more of the awareness out nowadays through the social media.  Participant-101: ...we need, like there's a Facebook out there, how about a VA book, how about a place to go where I can, “Hey, I've got hepatitis C and now I’ve got birds of a feather flocking together” [...] Now we can communicate up and down, we can talk to Congress, Senate, the president, you, the researchers and each vet and say, “What’s our problem, what's our issues?” |
| **Policy Improvement:**  Access and Costs | Participant-102: … a homeless person maybe they need transportation, they can try and get a grant so they at least have money to pay a driver to bring them to their appointments because they really want that treatment.  Participant-102: … when you go to transition out of the military, you have to have a physical. They could actually do a blood test then, and then when you get to wherever you’re going to live, the VA that you end up initially going to, when you have your first physical, they could do a blood test, too.  Participant-106: ... they should give us transportation.  Participant-106: You have yellow cabs, why not have Veteran cabs?  Participant-106: I think that they should have a visiting nurse for certain situations that would administer the medication.  Participant-118: … maybe the VA could just possibly pass out bus passes or something on that line….  Participant-118: Well, he was just talking about the blood work, isn’t that something that’s easy to do? How many times you go in and they want to test you for this and this and check this? Why don’t they just add that too it? Is it that what you’re talking about an easy way to overcome some of this? Am I off base? Participant-103: No, you’re right on point. […] Participant-106: I think that what they're saying it should be a mandatory thing since it’s such a silent killer.  Participant-104: … the government could override pharmaceutical companies on these prices.  Participant-104: I think shuttle services should be more accessible and maybe like the mass transit, it’s pretty accessible but, yeah, and veterans should be, you know, give them a bus pass so they can get to the VA.  Participant-104: I think it would be more cost effective to provide the ride to get people in for their medication than it would be to send a nurse, that would, it would cost three times as much at least.  Participant-108: First off, if you wanted to get this whole thing straightened out completely, it should be part of the ETS out of the service. There should be an automatic blood test before you can even get out...  Participant-107: I think the blood test for every new patient would be the easiest way to screen them out for any of their health problems. Participant-111: Right, both at the end of the Army and when they enter the VA. Participant-110: I agree with you, I think when you enter the VA healthcare system, the first thing they usually do is give you a CBC, a broad-spectrum blood test and I think the Hepatitis C test should be included.  Participant-107: Yeah, the government should have patent infringement on the drugs that help this big amount of the population. [...] Participant-110: Yeah, because I was amazed when I got the Blue Cross bill for $41,000 for 120 pills, well and a couple of face-to-face and some blood tests, I thought that cost was out-of-the-box. [...] Participant-107: Well the pharmaceuticals, they’ll get recuperative funding until all their technology, all their investigation and studies and everything, until their paid for, the cost of that will not change.  Participant-111: I think by testing veterans like that they can catch these diseases earlier and it'll be cheaper to treat to start with, they won’t have no liver transplants and things and desperate veterans with multiple diseases to treat. Participant-110: I think you're absolutely right. What’s it called, preventative healthcare...  Participant-111: … the VA medical could make a deal with a pharmaceutical company to contract all of the medications from just one that they could get it at a lower price, and then they could treat more people, that is an alternative. I mean, I don't know to this, whether it could possibly treat more people and bring down the cost...  Participant-110: I say that if they start testing all people as they leave the military, prior to their discharge, they give them the diagnosis, do you have hepatitis C or not, okay, they have to follow up with testing all veterans that come into the system and trying to reach out to all veterans that haven’t come into the system to come into the system and get the test, and make the test mandatory when you enter the system.  Participant-115: The government regulates everything else, why don’t they regulate something like this? Because the pharmaceutical companies are so rich they have bought the politicians.  Participant-121: I was thinking of maybe having a mobile unit. [...] just like we have those who can’t make it to the dentist, that that unit comes to them and makes a schedule for those who need to be seen.  Participant-122: Why aren’t they investing in testing for active military and veterans? I mean, does that make sense? It should be something that becomes a standard operating procedure, test you before you come in, test you before you go out.  Participant-123: … if they had transportation available for those people that were going to go through the hepatitis C program…  Participant-125: Well that’s where the other programs come in for transportation, getting an advocate to say, “Hey, I’ll pick you up here on this corner at a certain time, certain day and get you in.”  Participant-126: Maybe it’s for our policy makers to finally stand up to the pharmaceutical companies to get the price even lower because this is just a price that the pharmaceutical companies set.  Participant-128: As far as treatment is concerned I think they should have more accessible, not hospitals, but clinics with full ways of testing different people for different diseases.  Participant-129: …why are they not screening vets when they’re discharged? We shouldn’t even be, it shouldn’t be part of a discussion. This should be, that’s what kind of irks me, makes me mad, okay? It should be a screening just like a lot of other screenings... |
| **Policy Improvement:**  Better Decision making | Participant-104: … there would have to be a panel [...] that would get checked off, yeah, that would be a check off, it would almost be like a point system…  Participant-103: I guess we need some kind of survey or something that people answer enough questions that will give you a ranking...  Participant-107: ...if there were 30 people here that was going to die in the next two months and stuff, I wouldn't want to be the healthy person there making the decision, I would like them to make it amongst themselves because they could figure out who's got a good life and grandchildren and this and that…  Participant-107: Maybe we could talk about that contractual letter, to send that out to all the veterans that might’ve been applied to this scenario, right, and give them the choice whether they want to come and get treated for it or not.  Participant-111: … But it might have to be a committee instead of an individual, a doctor might be saving all the certain kind of guys...  Participant-111: You make it work by having a grouping like this and you give these details points, age has 5 points to consider, behaviors of people that don’t look like they’re having a good quality of life to you, it’s a personal decision after all, another 5 points, other sicknesses another 5 points, give all these possible conditions points and the guy with the highest number of points wins or loses, depending on how you look at it.  Participant-112: If you add a modification then you would add a panel of doctors to determine who’s the sickest first. [...] Right you know, so there’s where your modification, you could have a panel of doctors to after you got a group of folks that you was ready to treat, then you have a group of professionals, doctors to actually determine who is the sickest.  Participant-113: I’m trying to say that the decision, the person who makes the decision of who is sickest should be done by more than just one person. In other words, he might recognize that this person is sicker than that one, but he take it to a group of people, 2 or 3 people and have a consult about it or something like that, just to make sure this person really is the sickest or whatever. I’m just saying because that could be his grandma over there, he might want to give her favoritism or whatever. I’m just saying, I know it’s silly, but it should be more than just one person making that decision.  Participant-114: I do believe the doctor plays a very important part in this where, I mean he knows who the sickest is and I think if somebody came that wasn’t as sick as that person, that they would intervene and say, “Hey, this person right here is sicker than you, why don’t you let them go on,” I believe anybody in the military would say yeah, I do believe that, but the doctor plays the most important part in that.  Participant-114: Again, I think that the doctor, professional people know about the sickest, the degree of sickness, I mean you know because I don't know, I’m not a doctor and I don't know anything like that, so I think that’s really in the doctor’s ballpark.  Participant-110: For every criteria you come up with, you come up with another criteria to modify that criteria because it’s like what’s sickest but you’ve got to consider the overall liver health or the age and lifestyle, their age, does it really matter? Participant-111: That’s why, you have to give these, I mean one way some clinics do is to give point values to all these and the highest point guy goes through.  Participant-110: ...a group of people is setting criteria for how to decide who gets it and when they get it and why they get it according to this criteria and we have just found out that it is not easy and that we cannot even come up with something that’s like 1-2-3 step…  Participant-101: That’s something that we can consider, put the sick people in a room and let them decide to the doctor, “I’m next,” he’s next after me and then... Participant-102: Well that and it works too because it’s like a group support. |
