# Supplementary material for: Eliciting patient views on the allocation of limited healthcare resources: a deliberation on hepatitis C treatment in the Veterans Health Administration
Source: BMC Health Serv Res. 2020 May 1;20:369. doi: 10.1186/s12913-020-05211-8 (PMC7193376; doi:10.1186/s12913-020-05211-8)
Supplement: Supplementary file 3 — Additional file 3. Semi-Structured Interview Guide. For preliminary interviews conducted to inform the design of deliberation materials. [file 12913_2020_5211_MOESM3_ESM.docx]

**Additional File 3: Semi-structured Interview Guide**

**Advanced Prediction Models to Optimize Treatment and Access for**

**Veterans with Hepatitis C and Liver Disease**

***IRB*** *– please note that we are conducting semi-structured interviews. This guide provides the interviewer possible questions and probes. With a semi-structured interview, the interviewer is open to change the wording of a certain question and possibly explore an idea the interviewee brings up during the interview. However, the interviewer will always guide the conversation back to the topics for which we are interested in obtaining knowledge from the interviewee.*

**Introduction**

Hello, Mr./Ms _____________________, this is ___________. I am calling on behalf of the VA for a research project you recently learned about while you were at an appointment at the VA.

I/One of my research team members spoke to you about doing an interview for a project called, “Treatment Access for Veterans with Hepatitis C & Liver Disease”. The interview should take approximately 30-45 minutes.

Is this still a good time to talk?

[If yes, continue; If no, reschedule. Up to 2 reschedule dates are allowed.]

Before we begin, I would like to take a minute to remind you why we are asking Veterans like you to participate in an interview. This interview will help us develop information and materials to help us to improve the way we discuss potential policies to treat Veterans with Hepatitis C. We are interviewing Veterans whether or not they have Hepatitis C.

Participation in this interview is purely voluntary. If at any time and for any reason, you would prefer not to answer any questions, please feel free not to answer. If at any time you would like to stop participating, please tell me. You will not be penalized in any way for deciding to stop participation at any time. What you say will not affect your care at the VA.

I will be taking notes during the interview but I would also like to record this interview to make sure that I remember accurately all the information you provide.

Any information you provide will be handled in a confidential manner. Only people working on this study will use the interview notes and recordings. We will take steps to ensure your answers stay confidential.

1. Transcribed interview – if we choose to get the recordings transcribed, your name will not appear on any of the transcripts. Each audio file will be labeled only with a study ID number. The file will be kept on a secure server..
2. Audio – this will be saved on our secure server and deleted off the recording device.
3. Interview notes – these will be typed up behind the VA firewall and saved in a folder separate from transcribed interviews

You will be given a $25.00 Meijer gift card as a small token of our appreciation for your assistance with our research project.

Do you have questions before we begin?

Are you still interested in participating in this interview?

*If no longer interested, say:*

Thank you very much for your time. Good-bye

If the participant agrees to participate, say:

Is it okay if I turn on the recorder now? [If agrees:] Before I ask my questions, I will say a few administrative details, and then I will begin.

*Turn DVR on and state the following:*

This is ________________[name of interviewer].

Today’s date is ____________

Time is _____________

Participant ID is ____________

I have a few general questions to begin:

1. What branch of the military did you serve?
2. How long did you serve?
3. What is the name of your VA primary care doctor?

For this next set of questions, we want to determine what you know about Hepatitis C. There are no right or wrong answers.

1. Would you please tell me what you know about Hepatitis C.

**Probes**:

- Tell me your understanding of how someone gets Hepatitis C.
- Tell me what your understanding is about symptoms and complications from Hepatitis C
- Have you ever known anyone with Hep C?

1. Tell me what you know about Hepatitis C treatment.

**Probes**:

- Tell me your understanding about how effective treatment is for Hepatitis C.
- Tell me your understanding about how well treatment is tolerated (i.e. side effects).
- Tell me your understanding about cost of Hepatitis C treatments.

1. Have you ever been diagnosed with, or told that you have Hepatitis C? (If “No”, Move to Access Questions)

- Who told you?
- How did that make you feel?
- What were you told about Hepatitis C and your treatment options?
- Have you ever received treatment for Hepatitis C?
  - - [If pt has had treatment] Please tell me about your treatment experience.
    - [If no treatment] Please tell me why you haven’t received treatment.
    - [If no knowledge of treatment – go on to next question]

**Access**

Before we continue, I want to read a brief description about Hepatitis C that was provided by a doctor. The reason we are sharing this information is so that everyone who we interview is hearing the same thing about Hepatitis C regardless how much they knew or don’t know. Also, I am not a doctor so I can’t answer questions you might have about Hepatitis C. If you have questions about Hepatitis C after our interview, I would recommend you talk to your doctor.

Here is a brief description about Hepatitis C and its treatment.

*Hepatitis C is a disease caused by a virus that infects the liver. It can affect someone’s health and how long they live. For example, you can get liver cirrhosis or liver cancer over a number of years without treatment. It is common for people to live with Hepatitis C for years without knowing they have it, because they do not have symptoms. So, most people diagnosed with Hepatitis C find out that they already have a long-term, chronic infection. There are new drugs that can treat most people with Hepatitis C.*

*These newer medicines almost always work to treat Hepatitis C, however, there are number of reasons why Veterans may not be able to receive treatment right away.*

I know that was a lot of information, can I repeat any of that for you?

Before we mention what some of the reasons are, we would like to know what *you* think some of the reasons are why Veterans may not receive treatment right away?

*Here’s some information about the newer medicines for Hepatitis C* *and some access issues/barriers related to Hepatitis C treatment.* *Again, this was written by a doctor.*

*Treatment of Hepatitis C is with a combination of medicines that can fight the viral infection and prevent serious liver problems like cirrhosis or liver cancer. They are used for 12 weeks to a year and help the body get rid of the virus. These newer medicines almost always work to treat Hepatitis C, but they have side effects and they cost a lot. Also, some people live far away, might not show up for a clinic appointment for various reasons, and there are not enough doctors to treat Hepatitis C.*

Can I repeat any of that for you?

Given what I just read about how Hepatitis C can be treated and the reasons why it is difficult to treat everyone right away, what do you think is the best way to approach treating patients with Hepatitis C?

***Probe***

*If states “Don’t Know” (see below)*

*If states, “The VA should get more money or resources”(see below)*

*If provides an answer – state: These are great ideas… (see below)*

Sometimes limited resources prevent us from treating everyone at the same time. Limited resources may include appointments to see doctors, money to treat everyone, not enough medications. How should we treat pts when we have limited resources?

Now, imagine you are someone in the VA that has to figure out the best policy to treat patients with Hepatitis C. For example, imagine there are 10 patients that have Hepatitis C and this year we only have resources to treat 2-3 patients. What process would you come up with to treat patients with Hepatitis C?

I’m going to describe a couple of policies that the VA ***could*** consider (note: these are NOT real) and I want to know your thoughts in each of the following situations.

- - One policy is that patients with the highest risk for getting sick [or the sickest patients] should get the treatment first.

***Probe***: What do you think of that policy? Why do you think that?

- - Another policy is first come, first served. Doctors treat patients with Hepatitis C in the order of when they are diagnosed with Hepatitis C.

***Probe***: What do you think that policy? Why do you think that?

1. Now you have heard about a couple different policies that the VA could consider for treating patients with Hepatitis C: first come, first served or treating pts at highest risk of getting sick first. Which policy do you think is the best policy?

***Probe (elicit reasons) -*** Some people think [opposite policy] is the best policy – how would you respond? [Or: Why do you disagree with them?]

1. After hearing these two potential policies, can you think of another policy?

***Probes:***

- - - Why do you think this may be a good/bad policy?
    - How do you think this policy would work? [Or how could we make this policy work?]
    - How would you rank this policy compared to the two policies discussed above (treating the sickest patients first or first come first serve)?

 Ok, I just have one more general question for you:

1. On a scale from 1-5, what is your satisfaction with the care you receive at your VA facility. A “1” is very unsatisfied and a “5” is very satisfied.
   1. Please explain why you gave this rating.

That is all the questions I have. Is there anything you would like to add that we didn’t discuss about Hepatitis C treatment?

Is there is anything else you would like to add?

Thank you very much for your time.

I will be mailing you a $25 gift card in the mail. Can I please verify your mailing address?

If you have any questions, you can call our study at [number] and ask for (Pia or Amanda). You may have to leave a voice mail, but someone should get back to you within 48 hours.

If they have any concerns about the VA, here is the contact info for the Patient Advocates:

Anwar [number]

Monique [number]

Phone hours: M-F 8:30 to 4 pm

Office hours: M-F 8:30 to 3:30 pm (you can ask the information desk)
