# Supplementary material for: Eliciting patient views on the allocation of limited healthcare resources: a deliberation on hepatitis C treatment in the Veterans Health Administration
Source: BMC Health Serv Res. 2020 May 1;20:369. doi: 10.1186/s12913-020-05211-8 (PMC7193376; doi:10.1186/s12913-020-05211-8)
Supplement: Supplementary file 4 — Additional file 4. Baseline Survey. Administered at the beginning of the day, before the start of the deliberation. [file 12913_2020_5211_MOESM4_ESM.docx]

Treatment and Access for Veterans with Hepatitis C

Additional File 4: Baseline Survey

**Survey Instructions**

This survey asks your opinions about treating Veterans with hepatitis C. We may use this information to make recommendations to the Department of Veterans Affairs on future decisions when treating Veterans with hepatitis C.

Please answer each question to the best of your ability. There are no right or wrong answers. You may also choose not to answer any question.

| **Knowledge of Hepatitis C and Treatment** |
| --- |

The **purpose** of these questions is to find out what you currently know about hepatitis C and its treatment. You may not know all the answers and that is okay.

1. Select one answer from each row.

|  | True | False | Don’t Know |
| --- | --- | --- | --- |
| 1. The liver is the body part most affected by Hepatitis C. |  |  |  |
| 1. Getting tattoos and piercings can put a person at risk for Hepatitis C. |  |  |  |
| 1. A vaccine (“shot”) can prevent Hepatitis C. |  |  |  |
| 1. Hepatitis C can be treated with medication. |  |  |  |
| 1. Once you've been treated for Hepatitis C, you can’t get it again. |  |  |  |
| 1. Hepatitis C may cause liver cirrhosis or liver cancer. |  |  |  |
| 1. Most people do not experience symptoms of Hepatitis C when they’re first infected. |  |  |  |

| **Your Views** |
| --- |

We cannot treat all Veterans with hepatitis C at the same time because there are **limited resources**. Examples of limited resources may include: not enough doctors, nurses, money, equipment, facilities, or medicines.

1. When there are limited resources, which of these policies for treating Veterans with Hepatitis C should the VA adopt? (choose one)

First come, first served -- Veterans are treated in the order in which they come in for treatment.

Sickest first – Veterans are treated in the order of how sick they are from Hepatitis C (eg. symptoms or complications such as liver cancer or cirrhosis).

2a.  On a scale of 1 to 10, how strongly do you feel about the option that you chose in question 2?

| Not strongly at all | | | | | Very strongly | | | | |
| --- | --- | --- | --- | --- | --- | --- | --- | --- | --- |
| 1 | 2 | 3 | 4 | 5 | 6 | 7 | 8 | 9 | 10 |

1. We have given you 2 different options for how to deal with limited resources when treating Veterans with hepatitis C (see #2 above). We may have overlooked an option that is better. If you have an idea for a better approach, please describe it in the space below:
2. Imagine you are a Veteran with hepatitis C. When there are limited resources, please indicate whether you would be willing to wait to be treated after another Veteran.

| **Would you be willing to wait to be treated…** | Yes, I am willing to wait | No, I am NOT willing to wait | Unsure |
| --- | --- | --- | --- |
| 1. …if another Veteran has more symptoms of hepatitis C? |  |  |  |
| 1. …if another Veteran has more complications (e.g., liver damage) from hepatitis C? |  |  |  |
| 1. …if another Veteran came in for treatment before you did? |  |  |  |
| 1. …if another Veteran is younger than you are? |  |  |  |
| 1. …if another Veteran is older than you are? |  |  |  |
| 1. …if another Veteran has more dependents (e.g., children, a spouse or partner, elderly parents)? |  |  |  |
| 1. …if another Veteran has additional health problems (e.g. diabetes, heart disease)? |  |  |  |
| 1. …if another Veteran *got* Hepatitis C because of high risk behavior (e.g., from IV drug use)? |  |  |  |
| 1. …if another Veteran is at higher risk for getting Hepatitis C again after already being successfully treated (e.g. is an IV drug user)? |  |  |  |
| 1. …if another Veteran is not good about taking necessary medications? |  |  |  |
| 1. …if another Veteran regularly misses appointments? |  |  |  |
| 1. …if another Veteran is homeless? |  |  |  |
| 1. …if another Veteran got hepatitis C because of military service? |  |  |  |

1. When there are limited resources to treat hepatitis C, do you think *policy makers* should consider the following factors?

| **Should policy makers consider…** | Yes, should be considered | No, should not be considered | Unsure |
| --- | --- | --- | --- |
| 1. …how ill the Veteran is with hepatitis C (sickest patients are treated first)? |  |  |  |
| 1. …when the Veteran came in for treatment (first come, first served)? |  |  |  |
| 1. …age of the Veteran? |  |  |  |
| 1. …whether the Veteran has dependents (e.g., children, a spouse or partner, elderly parents)? |  |  |  |
| 1. …whether the Veteran has additional health problems (e.g., diabetes, heart disease)? |  |  |  |
| 1. …whether the Veteran *got* Hepatitis C because of high risk behavior (e.g., from IV drug use)? |  |  |  |
| 1. …whether the Veteran is at high risk for getting Hepatitis C again (e.g., is an IV drug user)? |  |  |  |
| 1. …whether the Veteran is good about taking necessary medications? |  |  |  |
| 1. …whether the Veteran regularly misses appointments? |  |  |  |
| 1. …whether the Veteran is able to pay for treatment? |  |  |  |
| 1. …whether the Veteran is homeless? |  |  |  |
| 1. …whether the Veteran got hepatitis C because of military service? |  |  |  |
| 1. …whether the Veteran has transportation to get to their treatment appointments? |  |  |  |
| 1. …whether the Veteran has social support? |  |  |  |

1. When there are limited resources, which Veterans with hepatitis C should be treated **first**? (choose one)

Veterans who have liver cancer.

Veterans who have liver cirrhosis but do not have liver cancer.

Veterans who have symptoms, but do not have liver cirrhosis or liver cancer.

Veterans who do not have symptoms.

All Veterans should be treated without delay regardless of symptoms or whether they have cirrhosis or cancer.

| **Your Views on the VA** |
| --- |

1. Where do you receive the majority of your health care?

VA

Non-VA

1. How satisfied are you with the care you receive at your VA facility?

Very satisfied

Satisfied

Neither

Dissatisfied

Very dissatisfied

[See next page]

1. The next questions are about your opinion of the **VA Healthcare system**, in general. For each statement below, please check how strongly you agree or disagree.

|  | Strongly Disagree | Disagree | Neutral | Agree | Strongly Agree |
| --- | --- | --- | --- | --- | --- |
| - 1. The VA Healthcare System does its best to make patients’ health better. |  |  |  |  |  |
| - 1. The VA Healthcare System covers up its mistakes. |  |  |  |  |  |
| - 1. Patients receive high quality medical care from the VA Healthcare System. |  |  |  |  |  |
| - 1. The VA Healthcare System makes too many mistakes. |  |  |  |  |  |
| - 1. The VA Healthcare System puts saving money above patients’ needs. |  |  |  |  |  |
| - 1. The VA Healthcare System gives excellent medical care. |  |  |  |  |  |
| - 1. Patients get the same medical treatment from the VA Healthcare System, no matter what the patient’s race or ethnicity. |  |  |  |  |  |
| - 1. The VA Healthcare System lies to make money. |  |  |  |  |  |
| - 1. The VA Healthcare System experiments on patients without them knowing. |  |  |  |  |  |

1. The next questions are about your opinion of your **VA primary care team**. For each statement below, please check how strongly you agree or disagree

|  | Strongly Disagree | Disagree | Neutral | Agree | Strongly Agree |
| --- | --- | --- | --- | --- | --- |
| - 1. I believe my VA PCP cares little about me. |  |  |  |  |  |
| - 1. My VA primary care team is usually considerate of my needs and puts them first. |  |  |  |  |  |
| - 1. I trust my VA primary care team so much that I always try to follow their advice. |  |  |  |  |  |
| - 1. If my VA primary care team tells me something is so, then it must be true. |  |  |  |  |  |
| - 1. I sometimes distrust my VA primary care team’s opinion and would like a second one. |  |  |  |  |  |
| - 1. I trust my VA primary care team’s judgement about my medical care. |  |  |  |  |  |
| - 1. I feel my VA primary care team does not do everything they should for my medical care. |  |  |  |  |  |
| - 1. I trust my VA primary care team to put my medical needs above all other considerations when treating my medical problems. |  |  |  |  |  |
| - 1. My VA primary care team are experts in taking care of medical problems like mine. |  |  |  |  |  |
| - 1. I trust my VA primary care team to tell me if a mistake was made with my treatment. |  |  |  |  |  |
| - 1. I sometimes worry that my VA primary care team may not keep the information we discuss totally private. |  |  |  |  |  |

1. For each statement below, please check how strongly you agree or disagree.

|  | Strongly Disagree | Disagree | Neutral | Agree | Strongly Agree |
| --- | --- | --- | --- | --- | --- |
| - 1. I trust the VA Healthcare System to decide who gets treated **first** for Hepatitis C. |  |  |  |  |  |
| - 1. I trust VA *health care providers* (e.g. primary care doctor, liver doctor) to decide who gets treated first for Hepatitis C |  |  |  |  |  |

| **Demographic Questions** |
| --- |

1. Experience with Hepatitis C: (Choose one)

I have completed treatment for Hepatitis C.

I am currently being treated for Hepatitis C.

I have Hepatitis C, but I am not currently receiving treatment for it.

I do not have Hepatitis C, but I have a close friend of family member with Hepatitis C.

I do not have Hepatitis C and I do not have a close friend or family member with Hepatitis C.

Prefer not to answer

1. What is your gender? _______________
2. What is your age? ______ years old
3. Are you of Spanish, Hispanic, or Latino descent?

Yes

No

1. Which of the following best describes your ethnic background? (Choose one)

African American / Black

Asian / Pacific Islander

Caucasian / White

Native American

Multi-Ethnic

Other (please specify): _________________________________

Prefer not to answer

1. What is the last year of school that you completed?

8th grade or less

Some high school

High school graduate or GED

Some college or 2-year college degree

4-year college degree

More than a 4-year college degree

Prefer not to answer

1. What is your current marital or domestic status?

Married

Living with partner

Divorced or separated

Widowed

Never married

Prefer not to answer

1. We would like to get an estimate of your household income in the past 12 months before taxes. Was it… (Choose one)

Less than $5,000  $40,000 to $49,999

$5,000 to $7,499  $50,000 to $59,999

$7,500 to $9,999  $60,000 to $74,999

$10,000 to $12,499  $75,000 to $84,999

$12,500 to $14,999  $85,000 to $99,999

$15,000 to $19,999  $100,000 to $124,999

$20,000 to $24,999  $125,000 to $149,999

$25,000 to $29,999  $150,000 to $174,999

$30,000 to $34,999  $175,000 or more

$35,000 to $39,999  Prefer not to answer

1. Which of the following best describes your current employment status? (Check all that apply)

Working full time, 35 hours or more a week

Working part-time, less than 35 hours a week

Unemployed or laid off and looking for work

Unemployed, not interested in returning to work

Homemaker

In school

Retired due to pain

Retired not due to pain

Disabled, not able to work

Other (please specify): _________________________________

Prefer not to answer

1. In which branch of the military did you most recently serve? (Check all that apply)

Air Force

Army

Coast Guard

Marine Corps

Navy

Other (please specify): _________________________________

1. How long did you serve? __________ years
2. In which era(s) did you serve in the military? (Check all that apply)

World War II

Early Cold War Era (1945 to 1949)

Korean Conflict (1950 to 1953)

Cold War Era (post Korean War) (1954 to 1963)

Vietnam Era (1964 to 1975)

Late Cold War Era (1976 to 1991) (Grenada, Beirut, Libya, Panama)

Post-Cold War Era (1991 to 2001) (Persian Gulf War, Somalia, Haiti, Yugoslavia)

Post 9/11 or War on Terrorism (2001 to present)

1. What date did you complete this survey?

| _____/ | _____/ | ______ |
| --- | --- | --- |
| Month | Day | Year |

**Thank you for completing this survey!**
