# Supplementary material for: Eliciting patient views on the allocation of limited healthcare resources: a deliberation on hepatitis C treatment in the Veterans Health Administration
Source: BMC Health Serv Res. 2020 May 1;20:369. doi: 10.1186/s12913-020-05211-8 (PMC7193376; doi:10.1186/s12913-020-05211-8)
Supplement: Supplementary file 6 — Additional file 6. Facilitator Discussion Packet. Given to the small group facilitators to help them guide the discussions. [file 12913_2020_5211_MOESM6_ESM.docx]

# Additional File 6: Facilitator Discussion Packet

# Session Agenda

| **Time (approximate)** | **Activity & Topic** |
| --- | --- |
| 9:00 – 9:30 (30 min) | **Registration**   - Continental breakfast - Complete or turn in any missing forms (Informed Consent/HIPAA) - Complete Baseline Survey |
| 9:30 – 9:45 *(*15 min) | **Welcome**   - ***Maria*** - General overview of the day – restroom location, lunch, breaks, etc. - ***Akbar*** - Presentation: Deliberative Democracy (Why are we here? What are we going to do? What is a Deliberative Democracy?) |
| 9:45 – 9:55 (10 min) | **Introductions and Ice Breaker - Small Groups** |
| 9:55 – 10:25 (30 min) | **Large Group Session ONE**   - ***Lauren*** - Presentation: Introduction to Hepatitis C Virus and its Treatment (20 min) - Q&A (10 min) |
| 10:25 – 11:10 (45 min) | **Small Group Session ONE**  **Chris/Heather/Pia/Monica (backups Grace and Heather) – S. Atrium**  **Claire/Kerry/Lizzie/George – G063**  **Ray/Jordan/Laura/Lauren – G064**   - Discuss the presentation and things that can get in the way of treating Veterans   - What are some ways we could overcome these things?   - What things can happen right away because they are easy to accomplish?   - What things might be difficult but would be possible? |
| 11:10 – 11:55 (45 min) | **Lunch** |
| 11:55 – 12:55 (60 min) | **Large Group Session TWO**   - ***George -*** Presentation: Caring for Veterans with Hepatitis C in the VA (20 min)   - Q&A (10 min) - ***Monica*** - Presentation: Hepatitis C Treatment: 2 Models (“First Come, First Served”   and “Sickest First”) (10 min)   - - Q&A (10 min) - ***Akbar*** - Explanation of deliberation task (10 min) |
| 12:55 – 1:50 (55 min) | **Small Group Session TWO (Policy Session)**  **Chris/Heather/Pia/Monica (back ups Grace and Heather) – S. Atrium**  **Claire/Kerry/Lizzie/George – G063**  **Ray/Jordan/Laura/Lauren – G064**   - **Policy discussion—Part 1**    - Discussion of pros and cons of each policy (15 min)   - **Voting**: “First Come, First Served” or “Sickest First” (5 min) - **Policy discussion—Part 2 (30 min)**   - Imagine we are in charge of determining the order in which Veterans with hepatitis C are treated. How (if at all) would you modify your table’s preferred policy to improve it? Thinking back to our last discussion, what other reasons do you think should be taken into consideration?   - Work toward coming to an agreed upon policy for treating Veterans with hepatitis C, which will then be presented to the larger group. |
| 1:50 – 2:00 (10 min) | **Break** |
| 2:00 – 2:30 (30 min) | **Large Group Discussion**   - ***Maria introduces Ray; Ray moderates discussion*** - Facilitator from each table reports their preferred policies (and their reasoning) to the larger group. - The large group will then discuss the policies and will have the opportunity to ask clarification questions. |
| 2:30 – 3:00 (30 min) | **Post-Deliberation Survey**   - Facilitators distribute participants’ final surveys. When the participant turns in their completed survey, they will receive their gift cards. |
| 3:00 – 3:30 (30 min) | **Facilitator De-Brief - G063**   - Once surveys distributed and participants are set with you, please go across hall to de-brief session - ***Before you leave – please make sure you do not have any identifiable materials in your possession (e.g participant list)*** |

**Veterans Deliberative Democracy**

**Saturday, July 28, 2018**

| **Presentation:** | Welcome and Housekeeping Details |
| --- | --- |
| **Presenter:** | Maria Hughes - Study Team |

| **Presentation:** | Deliberative Democracy: Why are we here? What are we going to do?  What is a Deliberative Democracy? |
| --- | --- |
| **Presenter:** | Dr. Akbar Waljee |
|  |  |

# Introductions and Ice Breaker

# Small Groups

**Location: All small groups in South Atrium**

**Discussion**: Introductions and Ice-breaker

**Agenda**: 9:45 – 9:55 (10 min)

**Reminders for Facilitators:**

- Please make sure the recorders are working properly.
- DO NOT TURN RECORDER ON for this first discussion until *after* you verbally consent participants (*see instructions below).
- State your table number and session number when you start the recorder
- Let participants know they each have one minute
- This is just a time to say hi, we will have time later to talk in more depth

**Verbal Consent of participants*:**

- **PRIOR** to turning DVR on:
  - Suggested language: “As you all agreed to when you were recruited, we are recording today’s discussions as was mentioned in the Informed Consent document you signed. I want to make sure this is still OK with each of you.”

**Note:** if anyone declines recording, flag Maria or Pia to explain to Veteran that they can’t participate if they won’t have their voice recorded.

- Once everyone has given verbal consent, state the following:
  - Suggested language: “I am going to turn the recording device on and when I do, we are going to go around the table and you will state either your first name ***or*** your study ID and state that you are consenting to your voice being recorded in all of today’s discussions.”
- TURN DVR ON
  - Say verbatim **“**This is table number _______ and Small Group Introduction and Ice Breaker Session. I will go around the table and ask each participant to provide verbal consent that he or she is OK with their voice being recorded throughout all of today’s Deliberative Democracy session”

**Introductions and Ice Breaker:**

Things to say to Participants (not word-for-word):

- “This morning, we would like to take a few minutes to get to know one another. My name is _____________ and my role today is to help the group stay on schedule and on topic. We may not always be able to spend as long on a topic as the group would like, but please know that we respect your opinions and appreciate the time you have volunteered to this study.”
- “As a reminder, **please begin your comments by using your first name *or* study ID**
- “We are doing this so we can keep track of who is saying what throughout the discussion, otherwise, peoples’ voices can sound like one another on the recording, and this makes it difficult for our transcriptionist and study to accurately keep track of everyone’s individual opinions.”
- “To protect your privacy, it is important that you use only your first name *or* study ID. Our recordings will be kept confidential, the transcripts will be de-identified, and we will not use your name in anything we write about this session.”
- “I will also try to use your name *or* study ID when speaking with you directly, so please do not feel singled out if I say your first name or study ID often during the discussion. Your specific comments will never be released in conjunction with your name.”
- “The ground rules for today’s discussions are**:**
- Keeping focused on issue/task
- Having a respectful interchange of opinions, even if there are strong disagreements
- Everyone should have a voice in the discussions
- New and creative ideas are welcome”
- “First let’s go around the table and introduce ourselves; please say….
- Your name *or* study ID and then:
  - The military branch in which you served and during what era (e.g., Vietnam or Korean era)?
  - Why you chose to participate in this study?

**Large Group Session ONE**

**Agenda**: 9:55-10:25 (30 min)

Dr. Beste’s presentation

**Small Group Session ONE**

| **Room** | **Facilitators** | **Timekeeper** | **Expert**  (Grace Su and Heather McCurdy are backups) |
| --- | --- | --- | --- |
| South Atrium | Chris/Heather | Pia | Monica |
| G063 | Claire/Kerry | Lizzie | George |
| G064 | Jordan/Ray | Laura | Lauren |

**Agenda**: 10:25-11:10 (45 min)

**Primary Discussion Topics:**

What are some things that get in the way of treating Veterans with hepatitis C?

- Given these, what are some ways we could overcome these things?
- What things can happen right away because they are easy to accomplish?
- What things might be difficult but would be possible?

**Ready?**

- Remind participants to state their first name *or* study ID.
- TURN RECORDER ON
- State: **“**This is table number _______ and Small Group Session One

**Say to Participants (not word-for-word):**

- “We’ve just heard a lot of information about hepatitis C and its treatment. We realize these may be sensitive topics; we welcome all ideas. We would like you to think about what would be best for other patients and for the VA *as a whole*, in addition to your personal preferences. If you have any questions for the experts, let me know and I will have them to come to our table. We have about 45 minutes for this discussion.”
- Discussion topics:
- Given this information, we’d like to hear your thoughts about what things get in the way of treating Veterans with hepatitis C.” (probe for their reasons)
  - - - Given these, what are some ways we could overcome these things?
      - What things can happen right away because they are easy to accomplish?
      - What things might be difficult, and take longer, but would be possible?

**Other Discussion Topics (if time allows):**

- What did you think about the presentation? Was anything unclear?
- Would you be willing to delay your own treatment so that another Veteran could get treated first? If so, under what circumstances?

**Large Group Session TWO**

**Agenda**: 11:55-12:55 (60 min)

Drs. Ioannou, Konerman, and Waljee presentation

**Small Group Session TWO**

| **Room** | **Facilitators** | **Timekeeper** | **Expert**  (Grace Su and Heather McCurdy are backups) |
| --- | --- | --- | --- |
| South Atrium | Chris/Heather | Pia | Monica |
| G063 | Claire/Kerry | Lizzie | George |
| G064 | Jordan/Ray | Laura | Lauren |

**Agenda**: 12:55-1:50 (55 min)

**Overview**:

- Part 1 (20 min):
  - Pros and cons of each policy (15 minutes)
  - Voting: “First Come, First Served” or “Sickest First” (5 minutes)
- Part 2 (30min):
  - Modifying the policy
  - Road testing the table’s policy

**Ready?**

- Remind participants to state their first name *or* study ID.
- TURN RECORDER ON
- State: **“**This is table number___ and Small Group Session Two

**Part 1:**

- **Pros and cons of each policy (15 minutes)**

**Voting: “First Come, First Served” or “Sickest First” (5 minutes)**

**Say to Participants (not word-for-word):**

- “We’ve just heard a lot more details about hepatitis C and how it’s treated. Now we’d like to hear your thoughts about which of the following two policies you think the VA should use to help when deciding which Veterans with hepatitis C get treated first.”

If you have any questions for the experts, let me know and I will have them to come to our table. We have about 20 minutes for this part of the discussion.”

- “The two policies to consider are:
  - 1. **First Come, First Served:** which means that doctors treat patients with hepatitis C in the order they come in to the VA.
    2. **Sickest First:** which means that Veterans with hepatitis C with the highest risk for getting sick (or the patients who are already sick) get treated first.

We’ll discuss these and then vote on which policy you think would be best.” (probe for their reasons)

1. Hand out a voting slip to each participant (NOTE: each slip will include the participant’s ID); instruct participants to circle one option.
2. Collect and tally votes, report majority to table.
3. **Keep track of the voting slips (collect and store).**

**Part 2:**

**Modifying the Policy and road testing the table’s policy (30 minutes)**

**Say to Participants (not word-for-word):**

- “Now, let’s talk about how we might want to modify our preferred policy to see if we can improve upon it.
- Imagine we are in charge of determining the order in which Veterans with hepatitis C are treated. How (if at all) would you modify our preferred policy to improve it?
- Thinking back to our last discussion, what other reasons do you think should be taken into consideration?
- Let’s work toward coming to an agreed upon policy for treating Veterans with hepatitis C, which will then be presented to the larger group.

Once your table has 1 or 2 modified policies:

- “Now, let’s test the policy/policies that we just came up with. Imagine you work at the VA and it is your job to figure out the best policy to treat Veterans with hepatitis C. For example, imagine there are 10 patients who have hepatitis C and this year we only have resources to treat 2-3 patients.”
- “After we are done, I will present them in our next section to the larger group and we will have a discussion with everyone in the room.”

When reporting to larger group, please include:

- Where table started
- The discussion to get to modified policy
- Mention any strong opinions
- Ask table if anything was missed

**Large Group Discussion**

**Location: All small groups in South Atrium**

- The table facilitator from each table reports their preferred policy/policies (and the reasoning behind them) to the larger group.
  - Ray will be invited to present first
  - Ray will call each table to present
    - 5 minutes to present and Q&A
- The large group will then discuss the policies and will have the opportunity to ask questions.

**After Large Group Session:**

- Thank everyone for coming
- Distribute Post-Deliberation Survey to appropriate person based on Study ID
- Instruct participants that when they complete survey to turn it in to study team member and collect gift card
- Once Facilitator has collected their belongings, head to G063 for Facilitator Debrief
- ***Before leaving – please make sure you have returned all study materials with identifiable information***
