# Supplementary material for: Eliciting patient views on the allocation of limited healthcare resources: a deliberation on hepatitis C treatment in the Veterans Health Administration
Source: BMC Health Serv Res. 2020 May 1;20:369. doi: 10.1186/s12913-020-05211-8 (PMC7193376; doi:10.1186/s12913-020-05211-8)
Supplement: Supplementary file 7 — Additional file 7. Participant Discussion Packet. Given to participants to help them follow the discussions. [file 12913_2020_5211_MOESM7_ESM.docx]

# Additional File 7: Participant Discussion Packet

| **Time (approximate)** | **Activity & Topic** |
| --- | --- |
| 9:00 – 9:30  (30 min) | **Registration**   - Continental breakfast - Complete or turn in any missing forms (Informed Consent/HIPAA) - Complete Baseline Survey |
| 9:30 – 9:45  (15 min) | **Welcome**   - General overview of the day – restroom location, lunch, breaks, etc. - Presentation: Deliberative Democracy (Why are we here? What are we going to do? What is a Deliberative Democracy?) |
| 9:45 – 9:55 (10 min) | **Introductions (Small Groups)** |
| 9:55-10:25  (30 min) | **Large Group Session ONE**   - Presentation: Introduction to Hepatitis C Virus and its Treatment - Q&A |
| 10:25-11:10  (45 min) | **Small Group Session ONE**   - Discuss the presentation and things that can get in the way of treating Veterans   - What are some ways we could overcome these things?   - What things can happen right away because they are easy to accomplish?   - What things might be difficult but would be possible? |
| 11:10-11:55 (45 min) | **Lunch** |
| 11:55-12:55  (60 min) | **Large Group Session TWO**   - Presentation: Caring for Veterans with Hepatitis C in the VA followed by Q&A - Presentation: Hepatitis C Treatment: 2 Models (“First Come, First Served” and “Sickest First”) followed by Q&A - Explanation of deliberation task |
| 12:55-1:50  (55 min) | **Small Group Session TWO (Policy Session)**   - Policy discussion—Part 1   - Discussion of pros and cons of each policy   - Voting: “First Come, First Served” or “Sickest First” - Policy discussion—Part 2   - Imagine we are in charge of determining the order in which Veterans with hepatitis C are treated. How (if at all) would you modify your table’s preferred policy to improve it? Thinking back to our last discussion, what other reasons do you think should be taken into consideration?   - Work toward coming to an agreed upon policy for treating Veterans with hepatitis C, which will then be presented to the larger group. |
| 1:50-2:00 (10 min) | **Break** |
| 2:00-2:30  (30 min) | **Large Group Discussion**   - Facilitator from each table reports their preferred policies (and their reasoning) to the larger group. - The large group will then discuss the policies and have the opportunity to ask clarification questions. |
| 2:30-3:00  (30 min) | **Post-Deliberation Survey**   - Facilitators distribute participants’ final surveys. When participant turns in their completed survey, they will receive gift cards. |

**Veterans Deliberative Democracy**

**Saturday, July 28, 2018**

| **Presentation:** | Welcome and Housekeeping Details |
| --- | --- |
| **Presenter:** | Maria Hughes - Study Team |

| **Presentation:** | Deliberative Democracy: Why are we here? What are we going to do?  What is a Deliberative Democracy? |
| --- | --- |
| **Presenter:** | Dr. Akbar Waljee |
|  |  |

*Personal notes:*

**Introductions and Ice Breaker**

**Small Group**

Discussion will be recorded. Please ***state your first name or*** ***your study ID*** whenever you speak. This is a big help to our transcriptionist! (Thanks!)

Please share the following with your small group (one person at a time):

1. In what branch of the military did you serve and during what era (e.g., Vietnam or Korean era)?
2. Why did you choose to participate in this study?

**Large Group Session ONE**

| **Presentation:** | Introduction to Hepatitis C Virus and its Treatment and Q&A |
| --- | --- |
| **Presenter:** | Dr. Lauren Beste |
|  |  |

*Personal notes:*

**Small Group Session ONE**

Discussion will be recorded. Please ***state your first name or*** ***your study ID*** whenever you speak. This is a big help to our transcriptionist! (Thanks!)

**Primary Discussion Topics:**

What are some things that get in the way of treating Veterans with hepatitis C?

- Given these, what are some ways we could overcome these things?
- What things can happen right away because they are easy to accomplish?
- What things might be difficult but would be possible?

Please **share your reasoning behind your positions** and think about **what would be best for all patients and for the VA as a whole,** in addition to your personal preferences.

*Personal Notes:*

**Large Group Session TWO**

| **Presentation 1:** | Caring for Veterans with Hepatitis C in the VA |
| --- | --- |
| **Presenter:** | Dr. George Ioannou |
| **Presentation 2:** | Hepatitis C Treatment: 2 Models  (“First Come, First Served” and “Sickest First”) |
| **Presenter:** | Dr. Monica Konerman |
| **Presentation 3:** | Explanation of Deliberation Task |
| **Presenter:** | Dr. Akbar Waljee |
|  | |

*Personal notes:*

**Small Group Session TWO**

Discussion will be recorded. Please ***state your first name or*** ***your study ID*** whenever you speak. This is a big help to our transcriptionist! (Thanks!)

Please **share your reasoning behind your positions** and think about **what would be best for all patients and for the VA as a whole,** in addition to your personal preferences.

**Definitions of each policy:**

- 1. **First Come, First Served:** Doctors treat patients with hepatitis C in the order of when they come in to the VA.
  2. **Sickest First:** Patients with hepatitis C with the highest risk for getting sick (or the patients who are already sick) get treated first.

**Discussion:**

- Part 1:
  - Pros and cons of each policy
  - Voting: “First Come, First Served” or “Sickest First”
- Part 2:
  - Imagine we are in charge of determining the order in which Veterans with hepatitis C are treated. How (if at all) would you modify your table’s preferred policy to improve it?
  - Thinking back to our last discussion, what other reasons do you think should be taken into consideration?
  - Work toward coming to an agreed upon policy for treating Veterans with hepatitis C, which will then be presented to the larger group.

*Personal notes:*

*Personal notes (continued):*

**Large Group Discussion**

- The table facilitator from each table reports their preferred policy/policies (and the reasoning behind them) to the larger group.
- The large group will then discuss the policies and will have the opportunity to ask questions.

*Personal notes:*

**Thank you for participating in the**

**Veterans Deliberative Democracy!**

- Complete Post-Deliberation Survey
- Turn Survey into Study Team member
- Collect gift card
